# Uncovering tomato quantitative trait loci and candidate genes for fruit cuticular lipid composition using the *Solanum pennellii* introgression line population

**DOI:** 10.1093/jxb/erx134

**Published:** 2017-05-04

**Authors:** Josefina-Patricia Fernandez-Moreno, Dorit Levy-Samoha, Sergey Malitsky, Antonio J Monforte, Diego Orzaez, Asaph Aharoni, Antonio Granell

**Affiliations:** 1 Fruit Genomics and Biotechnology Laboratory, Instituto de Biología Molecular y Celular de Plantas (CSIC-UPV), Ciudad Politécnica de la Innovación, Universidad Politécnica de Valencia, Av/ Ingeniero Fausto Elio s/n, CP, Valencia, Spain; 2 Department of Plant Sciences and the Environment, Weizmann Institute of Science, Ullmann Building of Life Sciences, Room, Rehovot, Israel; 3 Genomics in Plant Breeding Laboratory, Instituto de Biología Molecular y Celular de Plantas (CSIC-UPV), Ciudad Politécnica de la Innovación, Universidad Politécnica de Valencia, Av/ Ingeniero Fausto Elio s/n, CP, Valencia, Spain

**Keywords:** Cuticle, cuticular waxes, cutin monomers, introgression line population, tomato fruit, quantitative trait loci

## Abstract

The cuticle is a specialized cell wall layer that covers the outermost surface of the epidermal cells and has important implications for fruit permeability and pathogen susceptibility. In order to decipher the genetic control of tomato fruit cuticle composition, an introgression line (IL) population derived from a biparental cross between *Solanum pennellii* (LA0716) and the *Solanum lycopersicum* cultivar M82 was used to build a first map of associated quantitative trait loci (QTLs). A total of 24 cuticular waxes and 26 cutin monomers were determined. They showed changes associated with 18 genomic regions distributed in nine chromosomes affecting 19 ILs. Out of the five main fruit cuticular components described for the wild species *S. pennellii*, three of them were associated with IL3.4, IL12.1, and IL7.4.1, causing an increase in *n*-alkanes (≥C_30_), a decrease in amyrin content, and a decrease in cuticle thickness of ~50%, respectively. Moreover, we also found a QTL associated with increased levels of amyrins in IL3.4. In addition, we propose some candidate genes on the basis of their differential gene expression and single nucleotide polymorphism variability between the introgressed and the recurrent alleles, which will be the subjects of further investigation.

## Teaser

The genetic basis of tomato fruit variability in cuticular lipid composition is analyzed using the *Solanum pennellii* introgression line population. A QTL map for cuticular lipids and associated genes is presented.

## Introduction

Modern tomato (*Solanum lycopersicum*) cultivars are the result of several rounds of domestication and improvement (Frary *et al.*, 2010, Gur *et al.*, 2011; Blanca *et al.* 2012). Wild relatives of cultivated tomato have often been used in modern breeding as a source of genes for abiotic stress tolerance (Pineda *et al.*, 2012), disease resistance (Sandbrink *et al.*, 1995), and increased yield (Gur and Zamir, 2004). The wild species *Solanum pennellii* is particularly well adapted to conditions of extreme environmental stress (Yeats and Rose, 2013; Bolger *et al.*, 2014) and has been an important germplasm donor for *S. lycopersicum*. Special characteristics of the *S. pennellii* leaf cuticle, including those involved in regulating cuticle-related genes (Bolger *et al.*, 2014), enable it to adapt to these conditions. These characteristics probably make *S. pennellii* an ideal source of genes for cuticle reinforcement. One of the genetic resources most frequently used to identify genomic regions associated with quantitative traits of interest is the *S. lycopersicum* cv. M82 × *S. pennellii* interspecific introgression line (IL) population (Eshed and Zamir 1995). In this set of lines, the full genome of the wild species is represented as small introgressed fragments in 75 nearly-isogenic lines of the cultivar M82 genetic background. Of the more than 3000 quantitative trait loci (QTLs) reported (Alseekh *et al.*, 2013), nearly 2000 control fruit quality traits, for example, Brix, sugar content, volatiles, firmness, or flavonoid content (Causse *et al.*, 2002; Chapman *et al.*, 2012; Alseekh *et al.*, 2013). However, to date, the *S. pennellii* population has not been used to study QTLs involved in fruit cuticle lipid composition.

The cuticle has been described as the outermost layer covering all aerial plant organs. It is largely composed of acyl lipids, polyphenols, polysaccharides, and proteins (Riederer and Müller, 2006; Segado *et al.*, 2016*a*). The acyl lipids found in the cuticle include a small fraction of soluble waxes (Samuels *et al.*, 2008; Yeats and Rose, 2013) embedded in an insoluble matrix of the polymer cutin. Cutin represents the major component of the cuticle and is an amorphous, viscoelastic layer consisting of C_16_ and C_18_ polyhydroxy and epoxyhydroxy fatty acids (Pollard *et al.*, 2008; Beisson *et al.*, 2012). Additionally, plant cuticles can contain non-acyl lipid components such as pentacyclic triterpenols or polyphenols (Buschhaus and Jetter, 2011). All these compounds define a layered structure in which, schematically, the cuticle is placed on the top, the cell wall at the base, and an interconnected region with both type of polymers is found in between. This layered structure, which was described almost a half century ago and is extensively reviewed by Jeffree (2006), has been questioned in the past few years. Some scientists have started to consider the plant cuticle as a non-layered structure derived from a modified epidermal cell wall (Guzman-Puyol *et al.*, 2015; Segado *et al.*, 2016*a*, 2016*b*) and to study the interactions between the cuticle components from a new perspective. For example, in the embryo there were cell wall components, mostly polysaccharides and pectins, partially merged with the cutin matrix in an amorphous structure during early stages of development (Javelle *et al.*, 2011). It is accepted that the cuticular membrane is deposited on and within the cell wall scaffold, creating a single, efficient barrier that plays important roles in plant physiology, ecology, development, and fitness (Riederer and Müller, 2006; Hen-Avivi *et al.*, 2014; Martin and Rose, 2014). Hence, the study of the cuticle represents an interesting goal for breeding, considering that any modification in the cuticle will be related to important traits such as yield, by preventing water loss or pathogen infections (Hovav *et al.*, 2007; Isaacson *et al.*, 2009; Buxdorf *et al.*, 2014), and quality, by modifying glossiness or firmness (Domínguez *et al.*, 2011; Chapman *et al.*, 2012; Lara *et al.*, 2014).

In recent years, the tomato cuticle has received increased attention that has focused on molecular dissection (Bourdenx *et al.*, 2011, Girard *et al.*, 2012, Hen-Avivi *et al.*, 2014, Lashbrooke *et al.*, 2015), biomechanical properties (Dominguez *et al.*, 2011), water loss (Hovav *et al.*, 2007; Isaacson *et al.*, 2009), fruit cracking (Domínguez *et al.*, 2012), pathogen infection, and glossiness (Kunst and Samuels, 2009). Most of these studies resulted from the characterization of a small number of mutants and/or transgenic lines displaying different molecular, biomechanical, or visual phenotypes. However, the biosynthesis of cuticular lipids and their regulatory mechanisms remain unclear (Yeats and Rose, 2013; Hen-Avivi *et al.*, 2014; Martin and Rose, 2014). The use of genetic variability derived from wild tomato relatives or breeding/mapping populations to identify the genetic basis of natural variation in cuticle traits has been very limited. A number of tomato fruit cuticle studies in wild species (Yeats *et al.*, 2012) have revealed substantial variation in cuticle composition in genetically distant wild relatives, including *S. pennellii*. The availability of ILs, including global gene expression patterns for each population line, and the recent release of the *S. pennellii* genome constitute an excellent opportunity to investigate the genetic basis of tomato cuticle variability (Bolger *et al.*, 2014). Therefore, the aim of the present study was to screen QTLs involved in cuticular wax and cutin monomer composition in tomato fruit cuticle using the *S. pennellii* IL population resource, and to propose candidate genes associated with them.

## Materials and methods

### Plant material

We used the interspecific IL population derived from the cross between the cultivated tomato *S. lycopersicum* cv. M82 and the wild tomato *S. pennellii*, LA716. Of the 75 ILs (Eshed and Zamir, 1995), 72 were analyzed for cutin monomer composition (the missing lines were IL3.1, IL5.2, and the subILs 4.3.2 and 6.2.2) and 63 were studied for cuticular waxes (the excluded lines were IL1.2, IL3.1, IL3.2, IL4.4, IL5.3, IL5.5, IL7.3, and the subILs 2.4, 5.2, 6.2.2, 9.1.3, and 9.3.1). The population was cultivated in a single-block trial, each block consisting of four consecutive plants, except for M82, which consisted of 10 plants, under standard growth conditions (natural light and controlled temperature, 24 °C during the day and 18 °C at night) in a greenhouse during the 2010 summer season in Rehovot, Israel. Four fruits per plant were harvested at the red stage, except for lines producing small fruits, for which eight red fruits were collected. To validate the genetic effects, in 2011 a number of selected lines were grown at the same facilities and under the same conditions. In this case, plants were randomized in the greenhouse.

### GC-FID and GC-MS combined profiling for cuticular lipids and data analysis

An efficient method recently described for wax screening (Fernandez-Moreno *et al.*, 2016) was used to screen the *S. pennellii* IL population for variability of cuticular lipid composition in fruit. Cuticular waxes were extracted as described previously (Fernandez-Moreno *et al.*, 2016). Briefly, discs of ~19 cm^2^ from tomato fruit pericarp were excised from the equatorial region of each fruit and enzymatically treated to remove the non-cutinized cell wall components. Then, the material was dried for 2 days and then dipped twice into 4 ml chloroform for 30 s at room temperature to extract both epicuticular and major intracuticular waxes. The internal standard *n*-tetracosane (0.2 mg/ml) was added to each extracted sample. Next, dewaxed tissue discs were exhaustively delipidated in methanol:chloroform (1:1 v/v) for 15 days and then air-dried for 12 hours to proceed with the cutin extraction. To analyze cutin monomer variability, we used a well-known method that involves cutin depolymerization by a methyl-esterification reaction followed by cutin monomer extraction in an organic solvent (Franke *et al.*, 2005; Shi *et al.*, 2012; Lashbrooke *et al.*, 2015). In brief, a portion of the initial dewaxed sample (~13 cm^2^) was used for depolymerization of the cutin matrix by incubating the samples in 2 ml methanol/BF_3_ (Aldrich catalogue no.: B1252-250MLl) for 16 hours at 70 °C. Then, *n*-tetracosane was added to the reaction mix before adding 2 ml of saturated NaHCO_3_–water solution to stop the reaction. Next, cutin monomers were extracted in 2 ml chloroform three times, transferring the organic fraction to an empty vial each time. The pooled organic fraction was washed twice with 1 ml distilled water and the remaining water was removed by anhydrous Na_2_SO_4_ salt. Finally, extracts were concentrated to 1 ml using a stream of N_2_ gas and stored at −20 °C until further analysis.

Cuticular lipid profiling was performed by gas chromatography–flame ionization detector (GC-FID) using fruit tissue from three different plants per IL as previously described (Adato *et al.*, 2009). Additionally, a reference sample was prepared containing a mix of the extracts (1:1:1 v/v) from the three plants per IL and used to identify cuticular waxes and cutin monomers using gas chromatography–mass spectrometry (GC-MS) as described by Mintz-Oron *et al.* (2008) and Fernandez-Moreno *et al.* (2016). For the validation analysis of the best QTL obtained during the initial screening, a second-year analysis was performed on selected ILs using the more informative GC-MS technology to quantify the cuticular lipids instead of the GC-FID used in the first-year screening.

Wax constituents were identified in the total ion chromatogram using their Kovats indices and by comparing their mass spectra with those of authentic standards as well as data from the literature as detailed by Fernandez-Moreno *et al.* (2016). Compound quantification was calculated as the ratio of the peak area of each compound and that of the internal standard. These data were normalized on the basis of the total amount of internal standard (5 μg per mixture) and the total extraction area (18.84 cm^2^ for cuticular waxes and 12.56 cm^2^ for cutin monomers) to produce the final dataset (μg cm^−2^). Before statistical analysis, data were pretreated by centering $\left(\tilde{x}ij=xij-\bar{x}i\tilde{x}ij=xij-\bar{x}i\right)$, where *i*=1…*I* rows referring to the metabolites and *j*=1…*J* columns referring to the different ILs, and scaling using range scaling $\left(\tilde{x}ij=\frac{xij-\bar{x}i}{Max(xj)-Min(xj)}\tilde{x}ij=\frac{xij-\bar{x}i}{Max(xj)-Min(xj)}\right)$ to facilitate the observation of minor cuticular lipids from the total set of components (van den Berg *et al.*, 2006).

### Hierarchical clustering analysis

Phenotype variation in the *S. pennellii* IL population for both cuticular wax and cutin monomer metabolites was represented using a two-way hierarchical clustering analysis (HCA). A normalized, pre-treated (centered and range-scaled) dataset was used for the HCA. Statistical analysis was performed with JMP^®^ 10.0.0 software (SAS Institute Inc., 2012).

### QTL analysis and mapping

Data were recorded for each single plant and the mean was calculated for each IL and used for subsequent analysis. Plants were not considered as biological replicates as they were grown in the same block. Cuticular trait QTL analysis was conducted by single nucleotide polymorphism (SNP) marker–trait association by one-way ANOVA. A set of 216 Solcap_SNP genetic markers previously genotyped by Ofner *et al.* (2016), distributed across the 72 ILs (see Supplementary Table S1 at *JXB* online), were used. These genetic markers allowed the identification of a total of 72 bins, each defined by the recombination points in the IL collection. Significant bins were selected for marker genotype comparison by a Tukey’s honest significant difference test, adjusting the statistical threshold to declare significant effects to *α*=0.05/72, following Bonferroni correction.

For QTL verification, selected ILs containing *S. pennellii* introgressions on genomic regions associated with cutin/wax composition were evaluated in 2011. IL means were compared with the M82 control mean by a Dunnett’s or Student’s *t* test (*P*<0.05), depending on whether multiple mean or pairwise mean comparisons were performed, respectively; in addition, effects of IL, year, and the interaction IL × year were assessed by two-way ANOVA. Statistical analysis was done using JMP^®^ 10.0.0 software (SAS Institute Inc., 2012).

### Candidate gene selection

Candidate genes for each significant metabolite QTL (mQTL) were selected on the basis of: (i) the corresponding gene annotation being related to the biosynthetic process affecting the metabolite differentially accumulated in the QTL; (ii) the differential gene expression level in the IL of interest being consistent with its participation. Expression values were obtained from the transcriptome profiling of the same *S. pennellii* IL population in the experiment, available at http://ted.bti.cornell.edu/. In this experiment, RPKM (reads per kilobase per million mapped reads) values were obtained from this website and correspond to Illumina RNA-sequencing analysis on samples of red tomato fruits; and (iii) SNP differences between *S. lycopersicum* and *S. pennellii* producing non-conserved protein changes. To determine this, we used the *S. lycopersicum* mRNA sequence of each candidate gene to perform a BLASTx (translated nucleotide to protein database) search against the *S. pennellii* protein database (http://www.solgenomics.net/tools/blast/). The protein alignment resulting from this BLASTx was inspected carefully to identify non-conserved amino acid substitutions. Based on the above, we named candidate genes as any gene in the QTL interval that satisfied two or three of these criteria.

### Histological staining and electron microscopy

Thin sections of fixed and embedded pericarp fruit tissue samples were stained for lipids with Sudan IV as previously described (Isaacson *et al.*, 2009; Shi *et al.*, 2012). For transmission electron microscopy (TEM), 9 mm^2^ fruit exocarp pieces were fixed using paraformaldehyde, glutaraldehyde, and osmium tetroxide solutions, and embedded in EPON resin following the protocol developed at the Electron Microscopy Unit of the Weizmann Institute of Science (Rehovot, Israel), available online at the website of the Electron Microscopy Unit of the Weizmann Institute of Sciences http://www.weizmann.ac.il/Chemical_Research_Support/EM_Unit/links. Imaging was performed using a Tecnai T12 transmission electron microscope (FEI). Ultra-thin sections were also used for fluorescence microscopy (Nikon fluorescence microscope, Nikon Eclipse e-800, Japan) and to measure cuticle thickness using ImageJ software.

## Results

### Cuticular lipids identified in the IL population

**Fig. 1.**
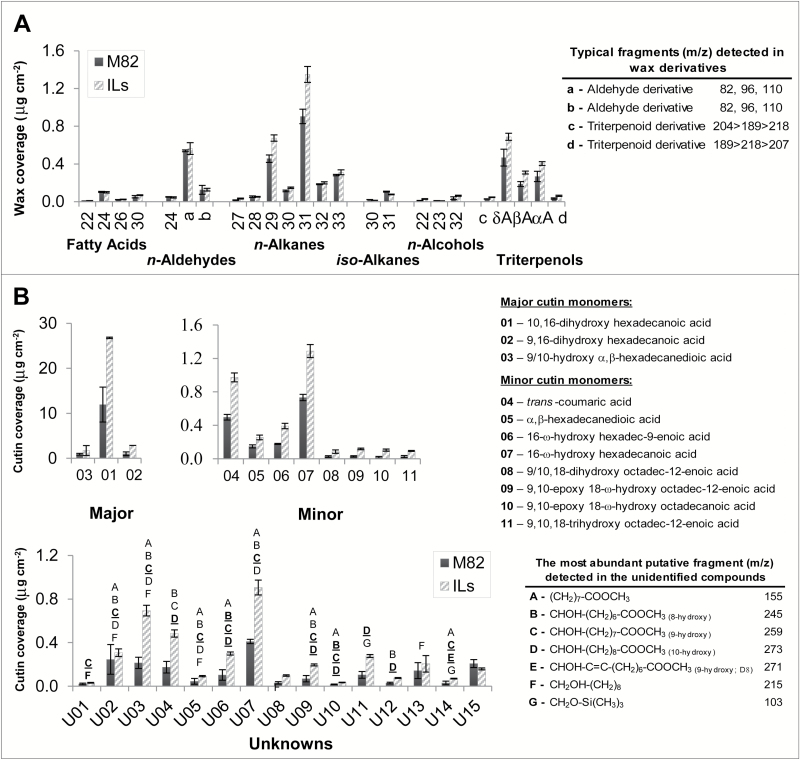
**Changes in cuticular lipid coverage in the *S. pennellii* IL population. A.** Wax coverage. **B.** Cutin monomer coverage. For the bars representing the parental *S. lycopersicum* cultivar M82, n=3; bars for ILs represent pooled data (n=63 and n=73 for wax and cutin monomer datasets, respectively). In A, aldehyde derivatives that were detected in the population are shown as a and b; triterpenol derivatives are shown as c and d. In B, uncharacterized cutin monomers are represented by ‘U’ and some of their structural information is provided. Data are presented as mean±SD.

A set of 24 cuticular waxes, including both epicuticular and the most abundant intracuticular waxes, was identified and used for the initial screening. The cuticular waxes identified and quantified were the following (Fig. 1A): even-numbered fatty acids C_22_, C_24_, C_26_, and C_30_; *n*-aldehyde C_24_; odd-numbered *n*-alkanes C_27_–C_33_ and even-numbered *n*-alkanes C_28_–C_32_; *iso*-alkanes C_30_ and C_31_; *n*-alcohols C_22_, C_23_, and C_32_; α-, β-and -amyrins (pentacyclic triterpenols). We detected a further two waxes with spectra similar to those of the *n*-aldehydes (labelled a, b in Fig. 1A) and others with spectra typical of pentacyclic triterpenoids (labelled c, d in Fig. 1A). The major waxes detected were the odd *n*-alkanes C_29_, C_31_, and C_33_, and the three amyrins (Fig. 1A).

For the cutin screening, a set of 11 cutin monomers was used (Fig. 1B): six C_16:X_ monomers (labelled #01–#03 and #05–#07 in Fig. 1B), including 10/9,16 dihydroxyhexadecanoic acid, the major cutin monomer accumulating in this population, (#01 in Fig. 1B); four C_18:X_ monomers (compounds #08–#11 in Fig. 1B); and one phenolic compound, *trans*-coumaric acid (#04 in Fig. 1B). An additional set of 15 cutin monomers was also detected (Fig. 1B). Their complete structures were not identified but they were characterized by the presence of structural features such as the 9(10)-midchain-hydroxyl group or some saturation. In total, a set of 50 cuticular lipid components (CLCs; i.e. 24 cuticular waxes and 26 cutin monomers) was detected in the IL population.

### Screening for cuticular lipid composition in the *S. pennellii* IL population

**Fig. 2.**
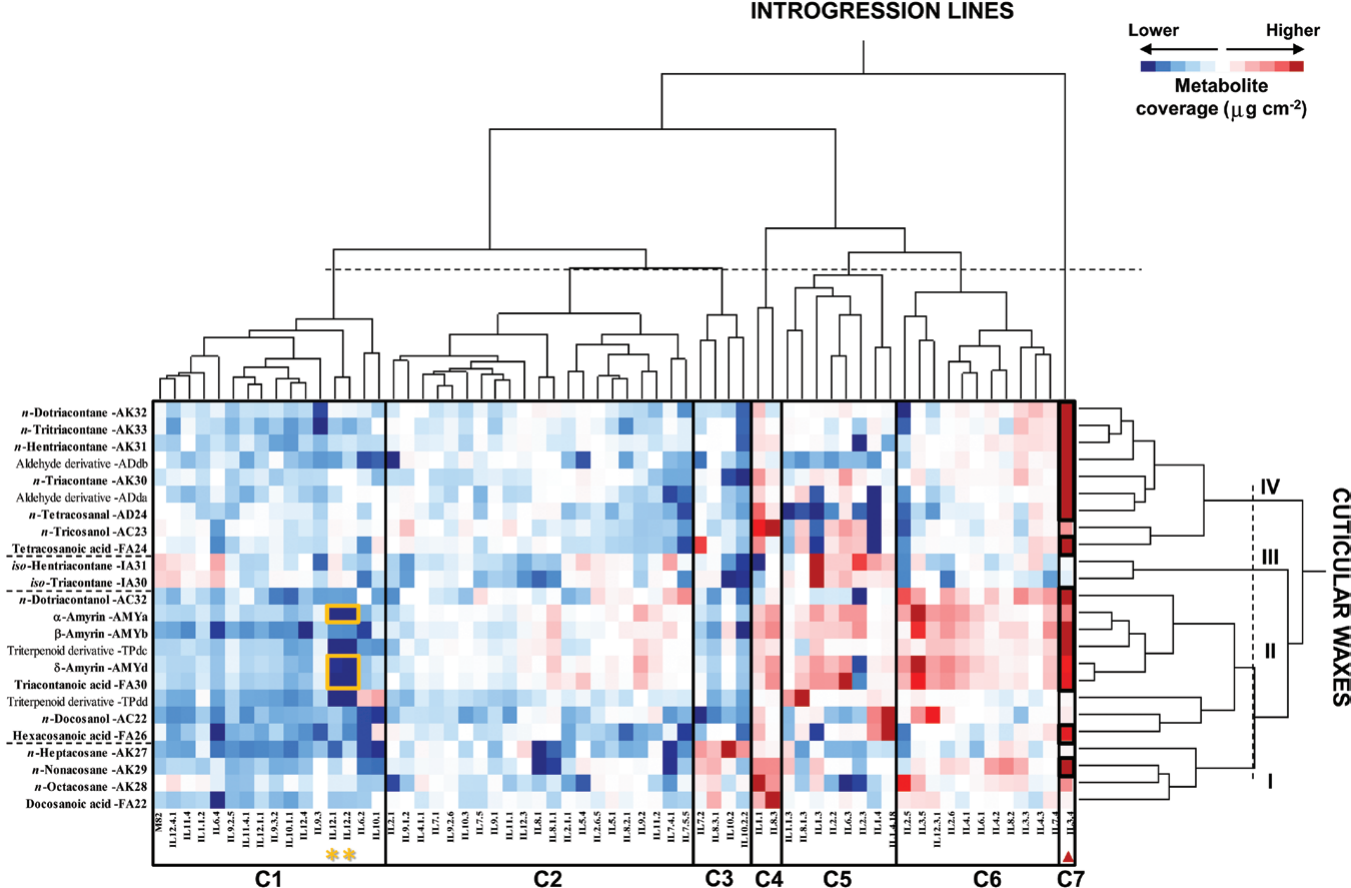
**Two-way hierarchical clustering heatmap of cuticular waxes and *S. pennellii* ILs**. The ILs were grouped into seven clusters (C1–C7); cuticular waxes were classified into four clusters (I–IV). Wax coverage (μg cm^−2^) for each compound in every line is represented by a colored gradient, the intensity of which ranges from dark blue, corresponding to lower wax accumulation levels, to dark red, corresponding to higher wax accumulation levels. The *vlcfa3.4* and *amy3.4* QTLs in IL3.4, containing cuticular waxes with different levels of accumulation relative to the parental *S. lycopersicum* M82 (black squares) in the second-year experiments (Student’s *t* test, *α*<0.05) are highlighted with a red triangle. Asterisks indicate the *amy12.1* QTL also described in previous reports (Yeats *et al.*, 2012; Bolger *et al.*, 2014; Ofner *et al.*, 2016). (This figure is available in colour at *JXB* online.)

**Fig. 3.**
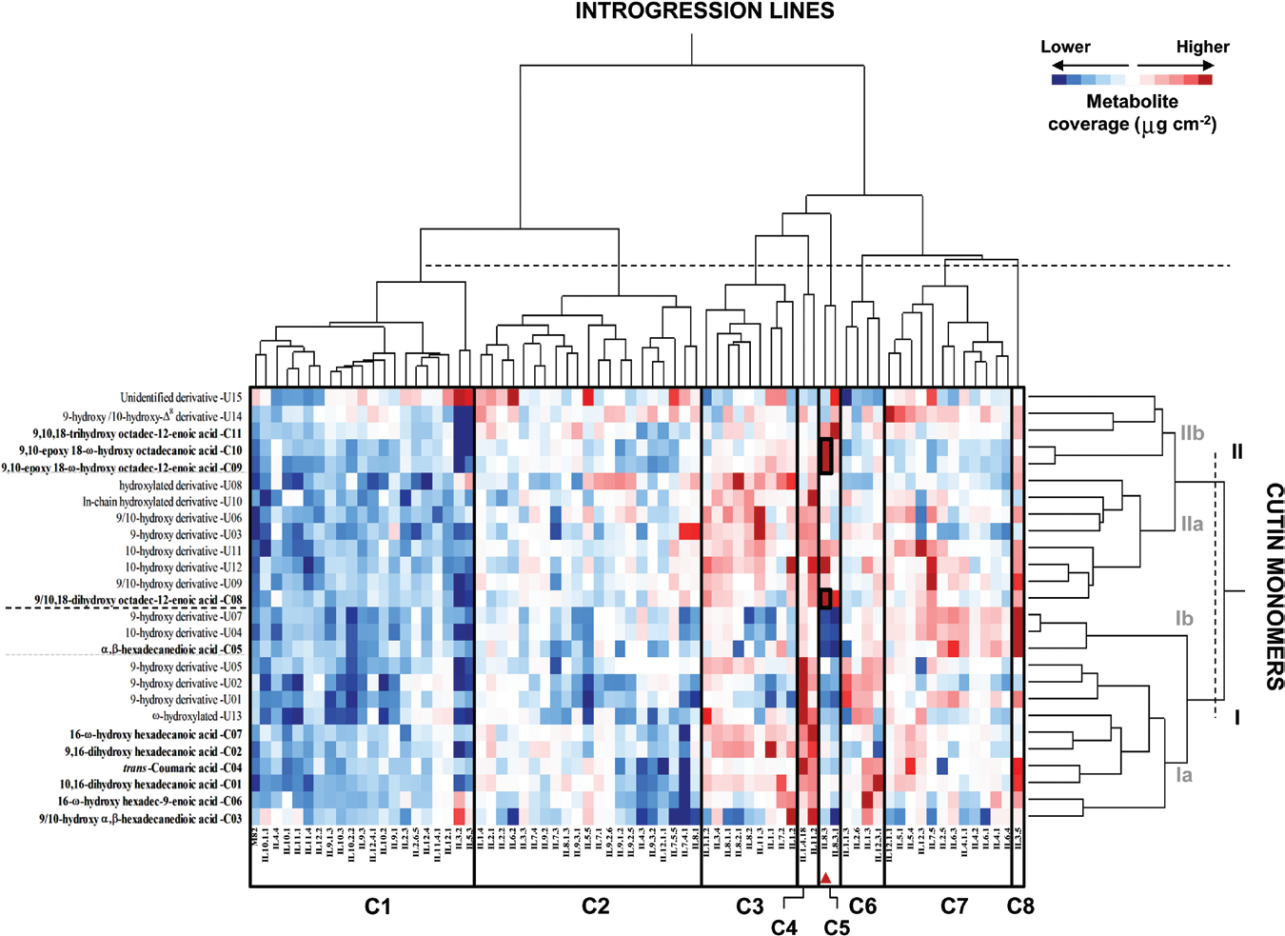
**Two-way hierarchical clustering heatmap of cutin monomers and *S. pennellii* ILs**. The ILs were classified into eight clusters (C1–C8); cutin monomers were classified into two main clusters (I and II) and four subclusters (Ia, Ib, IIa, and IIb). Cutin coverage (μg cm^−2^) for each monomer in every line is represented by a colored gradient, the intensity of which ranges from dark blue, corresponding to lower wax accumulation levels, to dark red, corresponding to higher wax accumulation levels. The *ehfa8.3* QTL in IL8.3 containing cutin monomers with different levels of accumulation relative to the parental *S. lycopersicum* M82 (black squares) in the second-year experiments (Dunnett’s test, *P*<0.05) is highlighted with a red triangle. (This figure is available in colour at *JXB* online.)

To determine the variability of the 50 CLCs across the population and the parental *S. lycopersicum* M82, two independent two-way hierarchical clustering analyses (HCAs) were performed, one for ILs and cuticular waxes (Fig. 2) and the other for ILs and cutin monomers (Fig. 3). The first HCA indicated that the ILs were grouped into seven clusters on the basis of wax coverage (Fig. 2), ranging from lines with lower wax levels in cluster C1 to those with higher levels in cluster C7. HCA results also showed that cuticular waxes could be organized according to the profiles obtained across the IL population, and this produced four clusters based on their abundance but also according to the class of wax. Thus, wax clusters II and IV comprised the most abundant cuticular waxes in the population and included amyrins and *n*-alkanes with more than C_30_ carbons, respectively, whereas wax clusters I and III contained the least abundant waxes and included *n*-alkanes with less than C_30_ carbons and *iso*-alkanes, respectively. In general, the IL population showed limited variability in fruit cuticular wax coverage (Fig. 2): just over half of the lines (58%) had similar wax coverage to the parental *S. lycopersicum* M82 (see clusters 1 and 2 in Fig. 2). The remaining lines showed higher differences in specific groups of waxes or, even, in particular wax compounds, when compared with the parental M82 (see clusters 3–7 in Fig. 2). For example, ILs in clusters C3 and C4 accumulated higher levels of *n*-alkanes with shorter chain length (<C_30_) than the rest of the population, whereas IL3.4 from cluster C7 accumulated higher levels of *n*-alkanes with more than C_30_ carbons. In addition, the triterpenoid wax class was found to be the most variable group in the entire population (see cluster II in Fig. 2), ranging from nearly no amyrin accumulation in IL12.1 and IL12.2 (in cluster C1) to almost a three-fold increase in amyrin levels in lines IL3.5 and IL3.4 (in clusters C6 and C7, respectively) when compared with the parental M82. In contrast, one of the least variable wax classes in the IL population was the *iso*-alkane class (see cluster III in Fig. 2). Here, the only exception was IL1.3 in cluster C5, which accumulated unusually high levels for both C_30_ and C_31_*iso*-alkanes by comparison with both the entire population and the parental M82.

HCA of cutin monomer composition organized the ILs into eight clusters (Fig. 3). ILs were grouped according to their cutin coverage, from lower levels in cluster C1 to higher levels in cluster C8 (Fig. 3). Interestingly, the 11 cutin monomers were grouped by their chain length, with C_16:X_ monomers in cluster I and C_18:X_ monomers in cluster II (Fig. 3). However, the subgrouping of two monomers—α,β-hexadecanedioic acid in cluster I and 9/10, 18-dihydroxy octadec-12-enoic acid in cluster II—differed from that of the other monomers in each respective cluster (see subclusters Ib and IIa in Fig. 3, respectively). The unidentified cutin monomers were also distributed across the four subclusters, probably by chain length and hydroxylation and/or saturation levels. For example, a putative mid-chain hydroxylated C_16:X_ monomer (labelled U13 in Fig. 3) was clustered together with other C_16:X_ monomers; and a putative 9,10,18-trihydroxy octadecanoic acid (U15) was grouped together with its Δ^12^-unsaturated counterpart, the 9,10,18-trihydroxy octadec-12-enoic acid in the same subcluster (Fig. 3). Based on the cutin monomer profiles, the IL population also showed cluster associations. In fact, five ILs displayed three clearly different patterns of cutin accumulation: IL3.5 over-accumulated mostly cutin monomers from cluster I (C_16:X_); IL8.3 and IL8.3.1 showed over-accumulation only of monomers belonging to cluster II (C_18:X_), particularly those in subcluster IIb; and IL1.4.18 and IL11.2 displayed over-accumulation of almost every cutin monomer in clusters I and II (Fig. 3).

### A draft cuticular lipid composition QTL map for tomato fruit in the *S. pennellii* IL population

**Fig. 4.**
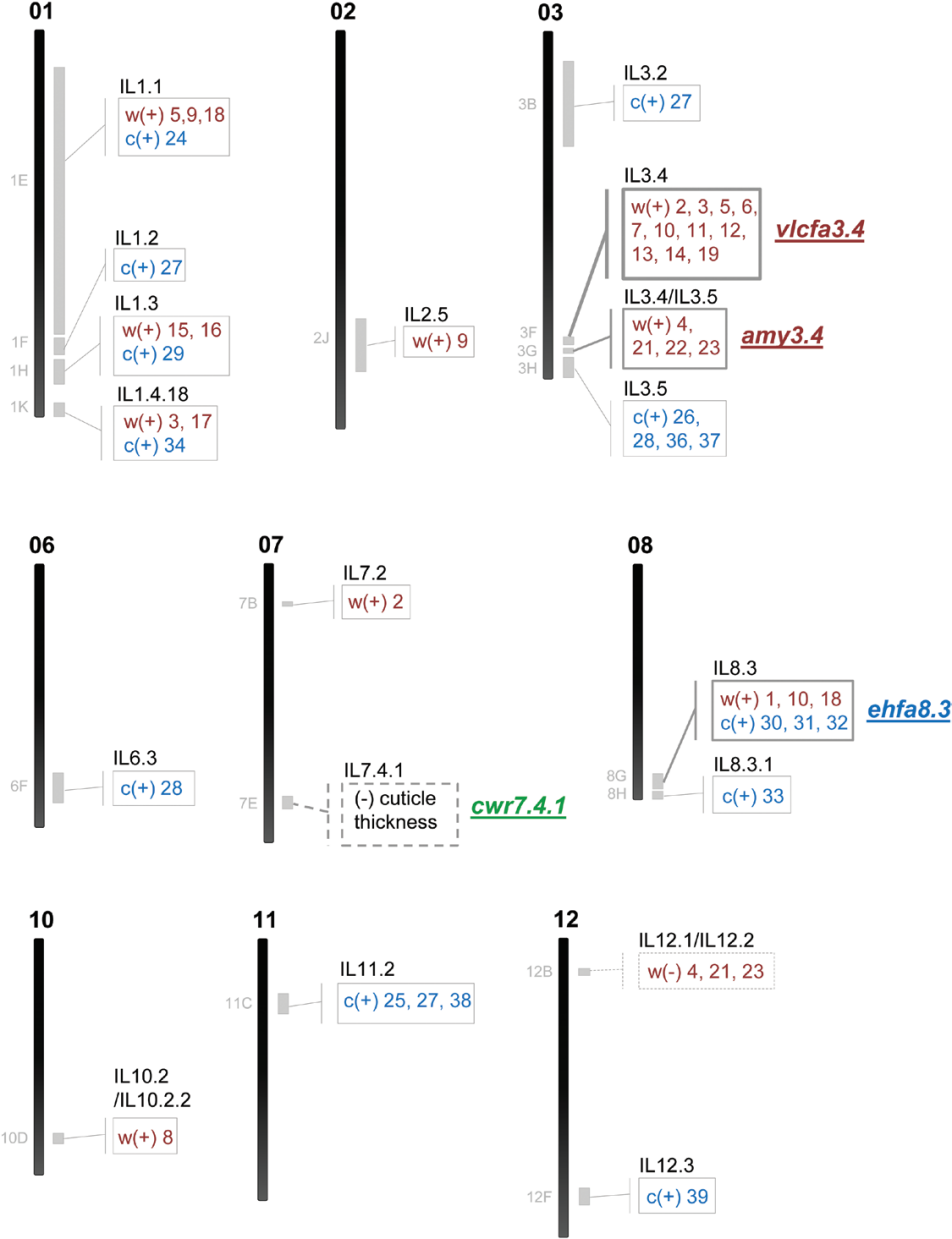
**QTL map for cuticular lipid composition in tomato fruit uncovered in the *S. pennellii* IL population.** The ILs containing significant changes for some cuticular lipids during the screening are indicated along the chromosomes, on the right side (see # numbers in Table 1). Waxes (w) are shown in red and cutin monomers (c) in blue. Most QTLs were positive (+) (cuticular lipid accumulation was higher in the IL than in the parental M82); negative (–) QTLs are represented with a dashed line in the map. The bins associated with the metabolic changes are also shown, on the left side of each chromosome (see Supplementary Table S1). The four QTLs confirmed by the second-year experiments (*vlcfa3.4*, *amy3.4*, *cwr7.4.1*, and *ehfa8.3*) are indicated on the map. See also details in Figs 5 and 6. (This figure is available in colour at *JXB* online.)

**Fig. 5.**
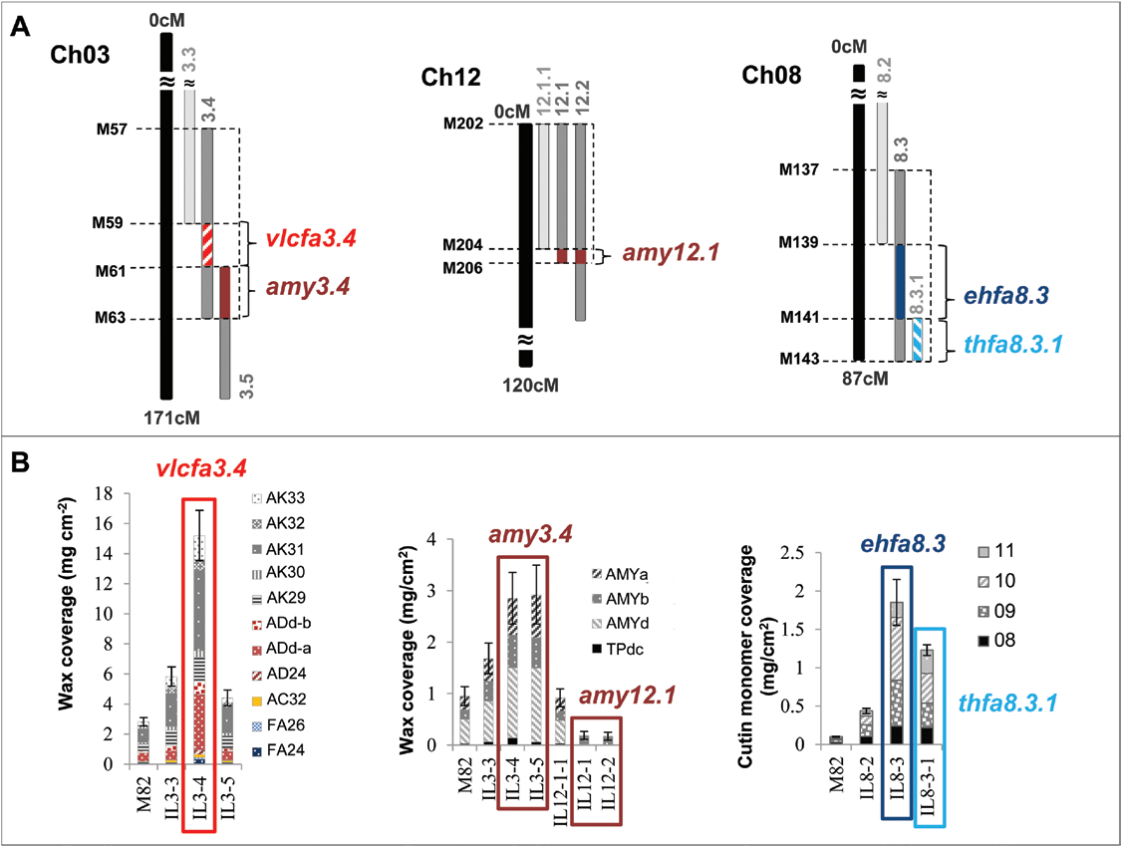
**QTLs associated with the main cuticular lipid traits of *S. pennellii* fruits**. **A.** Chromosome intervals. **B.** CLC metabolites with differential accumulation in association with each of the intervals in A. AC, alcohol; AD, aldehyde; ADd, aldehyde derivative; AK, alkane; amy, amyrins; ehfa, epoxyhydroxy fatty acids; FA, fatty acid; thfa, trihydroxy fatty acids; TPd, triterpenoid derivative; vlcfa, very-long-chain fatty acids. See cutin monomers #08–#11 in Fig. 1. For more genetic marker information, see Supplementary Table S1. Data are presented as mean±SD. (This figure is available in colour at *JXB* online.)

Cuticular waxes and cutin monomers were associated to SNPs by means of one-way ANOVAs using the IL genotype matrix from Ofner *et al.* (2016). SNPs located in nine different chromosomes (except chromosomes 4, 5, and 9), which were included in the introgressed *S. pennellii* regions of 19 ILs (~26% of the population), were associated to cuticular wax and/or cutin monomer variations. Table 1 summarizes the ILs containing genomic regions associated with differential metabolite accumulations; these metabolites are also mapped in Fig. 4 and indicated in Figs 2 and 3. Most of these genomic regions were associated with just one or two differentially accumulated cuticular lipid compounds. However, five of these genomic regions were associated with changes in a larger set of cuticular lipids. We focused on these five genomic regions, which were located in four ILs belonging to three chromosomes, affecting the composition of both cuticular waxes and cutin monomers. Accumulation of the major cuticular waxes detected in the population (i.e. *n*-alkanes with >C_30_ carbons in length and amyrins) was significantly altered in IL3.4 and IL12.1. Thus, whereas IL3.4 exhibited increased accumulation of both types of major cuticular waxes, IL12.1 showed reduced levels for just one type (amyrins). These changes were associated with three different genomic regions: two of them mapped to IL3.4 and the third to IL12.1 (Fig. 5A). The first of these regions mapped to a 600 kb interval in IL3.4 not shared by either IL3.3 or IL3.5 (Fig. 5B), which was associated with increased levels of *n*-aldehydes and *n*-alkanes (Fig. 5B). The second region in IL3.4 mapped to a 410 kb interval shared with IL3.5 (Fig. 5A) and also associated with increased levels of the three amyrins (Fig. 5B). The third region mapped to a 0.98 Mb interval in IL12.1 shared with IL12.2 (Fig. 5A), which was associated with a dramatic reduction in levels two of the three amyrins: δ- and α-amyrins (Fig. 5B). The metabolic phenotype observed in this last region has been reported previously in tomato fruit using both the *S. pennellii* and *Solanum habrochaites* IL populations (Yeats *et al.*, 2012), and more recently in tomato leaves using the same *S. pennellii* IL population (Bolger *et al.*, 2014; Ofner *et al.*, 2016). In fact, two *TRITERPENOID SYNTHASE* genes, *SlTTS1* and *SlTTS2* (*Solyc12g006530* and *Solyc12g006520*, respectively; Table 2, Supplementary Tables S2 and S3) involved in the conversion of epoxysqualene to amyrins and other triterpenoids were described in this interval. *SlTTS1* was described as a specific β-amyrin synthase, whereas *SlTTS2* seemed to be a general amyrin synthase, producing mostly δ-amyrin and then α-amyrin (Wang *et al.*, 2011; Yeats *et al.*, 2012). As the metabolic phenotype for neither of the other two regions in IL3.4 had been reported previously, we wanted to check whether they would be also a consistent QTL. With the purpose of validating these two QTLs, IL3.4 was analyzed and evaluated in a second experiment the following year. Similar to what we observed in the first season, the three amyrins and most of the *n*-alkanes and *n*-aldehydes were differentially over-accumulated in IL3.4 (Supplementary Fig. S1A). On the basis of these results, two wax-related QTLs in IL3.4, a very-long-chain fatty acid QTL (*vlcfa3.4*) and an amyrin QTL (*amy3.4*), were defined.

**Table 1.** QTLs for cuticular lipid composition in tomato fruit uncovered in the S. pennellii introgression line population

#	Chain length	Other name	M82sp^*a*^	Introgressed region affected^*b*^
Waxes				
Fatty acids				
1	22		0.007 ± 0.0008	IL8.2/IL8.3 | IL8.3 (0.04 ± 0.034) | IL8.3.1 (0.02 ± 0.015)
2	24		0.1 ± 0.01	IL3.4 (0.3 ± 0.11) | IL7.2 (0.3 ± 0.13)
3	26		0.02 ± 0.002	IL1.4.18 (0.1 ± 0.01) | IL3.3/IL3.4 (0.1 ± 0.02)
4	30		0.05 ± 0.021	IL3.4 (0.1 ± 0.02)/IL3.5 (0.1 ± 0.03) | **IL12.1** (ND)/**IL12.2** (0.005 ± 0.0001)↓
*n*-Aldehydes				
5	24		0.05 ± 0.007	IL1.1 (0.1 ± 0.02) | IL3.3/IL3.4 (0.3 ± 0.12) | IL3.4 | IL3.4/IL3.5
6		derivative a	0.5 ± 0.02	IL3.3/IL3.4 (3.7 ± 1.68) | IL3.4 | IL3.4/IL3.5
7		derivative b	0.1 ± 0.08	IL3.3/IL3.4 (1.0 ± 0.28) | IL3.4 | IL3.4/IL3.5
*n*-Alkanes				
8	27		0.02 ± 0.005	IL10.2 (0.2 ± 0.09) | IL10.2.2 (0.1 ± 0.01)
9	28		0.05 ± 0.015	IL1.1 (0.2 ± 0.06) | IL2.5 (0.2 ± 0.02)
10	29		0.5 ± 0.07	IL3.4 (1.6 ± 0.29) | IL8.2 (1.3 ± 0.77) / IL8.3 (1.3 ± 0.82)
11	30		0.1 ± 0.01	IL3.4 (0.4 ± 0.09)
12	31		0.9 ± 0.13	IL3.4 (5.3 ± 1.97)
13	32		0.2 ± 0.01	IL3.4 (0.7 ± 0.28)
14	33		0.3 ± 0.01	IL3.3 (0.8 ± 0.30)/IL3.4 (1.6 ± 0.70)
*iso*-Alkanes				
15	30		0.02 ± 0.002	IL1.3 (0.04 ± 0.001) | IL1.3/IL1.4
16	31		0.1 ± 0.01	IL1.3 (0.2 ± 0.01) | IL1.3/IL1.4
*n*-Alcohols				
17	22		0.01 ± 0.002	IL1.4.18 (0.2 ± 0.01)
18	23		0.01 ± 0.002	IL1.1 (0.02 ± 0.011) | IL8.3 (0.02 ± 0.018)
19	32		0.04 ± 0.025	IL3.3 (0.2 ± 0.02)/IL3.4 (0.2 ± 0.05) | IL3.4 | IL3.4/IL3.5 (0.1 ± 0.03)
Triterpenols				
20		derivative c	0.03 ± 0.002	IL3.3 (0.06 ± 0.006)/IL3.4 (0.1 ± 0.01) | IL3.4 | IL3.4/IL3.5 (0.06 ± 0.052)
21		δ-Amyrin	0.5 ± 0.16	IL3.4 (1.4 ± 0.024)/IL3.5 (1.4 ± 0.032) | **IL12.1** (0.01 ± 0.002)/**IL12.2** (ND)↓
22		β-Amyrin	0.2 ± 0.05	IL3.4 (0.6 ± 0.06)/IL3.5 (0.6 ± 0.20)
23		α-Amyrin	0.3 ± 0.09	IL3.4 (0.7 ± 0.11)/IL3.5 (0.8 ± 0.20) | **IL12.1** (0.01 ± 0.001)/**IL12.2** (0.01 ± 0.006)↓
Cutin monomers				
Major cutin monomers				
24	16	9,16-dihydroxy hexadecanoic acid	1.0 ± 0.68	IL1.1 (7.7 ± 1.51)
25	16	9(10)-hydroxy α,β-hexadecanedioic acid	0.9 ± 0.36	IL11.2 (4.2 ± 0.71)
Minor cutin monomers				
26		*trans*-coumaric acid	0.5 ± 0.06	IL3.5 (2.4 ± 1.02)
27	16	α,β-hexadecanedioic acid	0.2 ± 0.03	IL1.2 (1.1 ± 0.74) | IL3.2 (1.0 ± 0.34) | IL11.2 (1.1 ± 0.04)
28	16	16-ω-hydroxy hexadec-9-enoic acid	0.2 ± 0.01	IL3.5 (1.5 ± 0.87) | IL6.3 (1.3 ± 0.92)
29	16	16-ω-hydroxy hexadecanoic acid	0.7 ± 0.07	IL1.3 (3.9 ± 2.61)
30	18	9,18-dihydroxy octadec-12-enoic acid	0.03 ± 0.017	IL8.3 (0.2 ± 0.09) | IL8.3.1 (0.2 ± 0.09)
31	18	9,10-epoxy 18-ω-hydroxy octadec-12-enoic acid	0.03 ± 0.012	IL8.3 (0.6 ± 0.16) | IL8.3.1 (0.3 ± 0.18)
32	18	9,10-epoxy 18-ω-hydroxy octadecanoic acid	0.02 ± 0.018	IL8.3 (0.8 ± 0.19) | IL8.3.1 (0.4 ± 0.26)
33	18	9,10,18-trihydroxy octadec-12-enoic acid	0.03 ± 0.024	IL8.3 (0.2 ± 0.04) | IL8.3.1 (0.3 ± 0.05)
Unidentified cutin monomers				
34		Unknown 02	0.3 ± 0.24	IL1.4.18 (1.6 ± 0.26)
35		Unknown 03	0.2 ± 0.09	IL11.2 (1.6 ± 0.54)/IL11.3 (2.1 ± 0.44) | IL11.3
36		Unknown 04	0.2 ± 0.09	IL3.5 (1.7 ± 0.86)
37		Unknown 07	0.4 ± 0.03	IL3.5 (3.3 ± 1.71)
38		Unknown 10	0.02 ± 0.005	IL11.2 (0.1 ± 0.02) | IL11.2/IL11.3
39		Unknown 11	0.1 ± 0.05	IL12.3 (0.3 ± 0.05)

^*a*^ Wax coverage (μg cm^−2^) and standard deviation (n=3) are provided for parental M82sp.

^*b*^ wax coverage (μg cm^−2^) and standard deviation (n=3) are provided for those lines showing values significant (*P*<0.0005) by one-way ANOVA.

ND Non-detected metabolites.

Only negative QTLs are in bold type and indicated with an arrow (↓).

**Table 2.** Candidate genes proposed for the five QTLs associated with the three of the main traits in S. pennellii fruit cuticle

QTL^*a*^	ILs	Chr	Bin (Mb)^*b*^	Phenotype	Gene ID	Annotation^*c*^
*Waxes*						
*vlcfa3.4*	IL3.4	3	M60^*d*^ (0.68)	Increased VLCFAs from decarbonylation pathway	*Solyc03g117800*	Homologous to *AtCER3* (*ECERIFERUM 3*)
*amy3.4*	IL3.4/IL3.5	3	M61–M63 (0.41)	Increased α-, β- and δ-amyrins	*Solyc03g118540*	Homologous to *AtJAZ3* (*JASMONATE-ZIM-DOMAIN PROTEIN 3*)
*amy12.1*	IL12.1/IL12.2	12	M205–M206 (0.98)	Decreased δ- and α-amyrins	*Solyc12g006530*	*SlTTS1* (*TRITERPENOID SYNTHASE 1*)
					*Solyc12g006520*	*SlTTS2* (*TRITERPENOID SYNTHASE 2*)
*Cutin monomers*						
*ehfa8.3*	IL8.3	8	M140^*e*^ (0.54)	Increased epoxyhydroxy fatty acids	*Solyc08g081220*	Homologous to *AtCYP86A8/LCR* (*LACERATA*)
*Cuticle thickness*						
*cwr7.4.1*	IL7.4.1	7	M122–M123 (2.18)	Reduced cuticle thickness	*Solyc07g056000*	Homologous to *AtXTR7*
					*Solyc07g055990*	Homologous to *AtXTR7*
					*Solyc07g056290*	Uncharacterized tomato *POLYGALACTURONASE 28*
					*Solyc07g055920*	Homologous to tomato *TAGL1* (*SHATTERPROOF 1*)

amy, amyrins; Chr, chromosome; ehfa, epoxyhydroxy fatty acids; thfa, trihydroxy fatty acid, vlcfa, very-long-chain fatty acids.

^*a*^ The IL number is included for each QTL.

^*b*^ Genetic markers are described in Supplementary Table S1. The size of the interval in million bases (Mb) is provided.

^*c*^ Gene annotations were obtained from the SL2.40 tomato database (http://www.solgenomics.net/).

^*d*^ The phenotype observed for *vlcfa3.4* was stronger in the non-overlapping interval of IL3.4. However, it was also observed for the overlapping regions IL3.3/IL3.4 (M57–M59) and IL3.4/IL3.5 (M61–M63).

^*e*^ The phenotype observed for *ehfa8.3* was stronger in the non-overlapping interval of IL8.3. However, it was also observed for the overlapping regions IL8.2/IL8.3 (M137–M139) and IL8.3/IL8.3.1 (M141–M143).

Most of the minor cutin monomers were found to be differentially accumulated in the IL population, but not the major cutin monomer, 10,16-dihydroxyhexadecanoic acid. Similar to what was observed for cuticular waxes, most of the ILs affected in their cutin monomer composition showed differential accumulation of one or two monomers. Only two genomic regions belonging to two overlapping ILs in chromosome 8 showed alterations in more than two cutin monomers (Table 1). The first of these regions mapped to a 540 kb interval in IL8.3 (Fig. 5A), which was associated with an increase in three C_18_ cutin monomers: 9,10-epoxy 18-ω-hydroxy octadecanoic acid, 9,10-epoxy 18-ω-hydroxy octadec-12-enoic acid, and 9/10,18-dihydroxy octadec-12-enoic acid (Fig. 5B). The second region mapped to a 360 kb interval shared by both IL8.3 and IL8.3.1 (Fig. 5A), which was associated with an increase in 9,10,18-trihydroxy octadec-12-enoic acid (Fig. 5B). In a similar way as for the promising regions in IL3.4, we also checked both IL8.3 and IL8.3.1 in a second-year experiment. In the second year, only two cutin monomers in IL8.3 were validated, while the other two were more variable for the parental M82 than in the first year. This explained why they were not significant enough in any of the IL8.3 and IL8.3.1 even when they were accumulated in higher levels in both ILs (Supplementary Fig. S1). Thus, we validated one of the two QTLs in chromosome 8: the epoxyhydroxy fatty acid QTL in IL8.3 (*ehfa8.3*). Further analysis should be carried out to further elucidate the trihydroxy fatty acid QTL in IL8.3.1 (*thfa8.3.1*).

### A QTL associated with cuticle thickness was mapped to chromosome 7

**Fig. 6.**
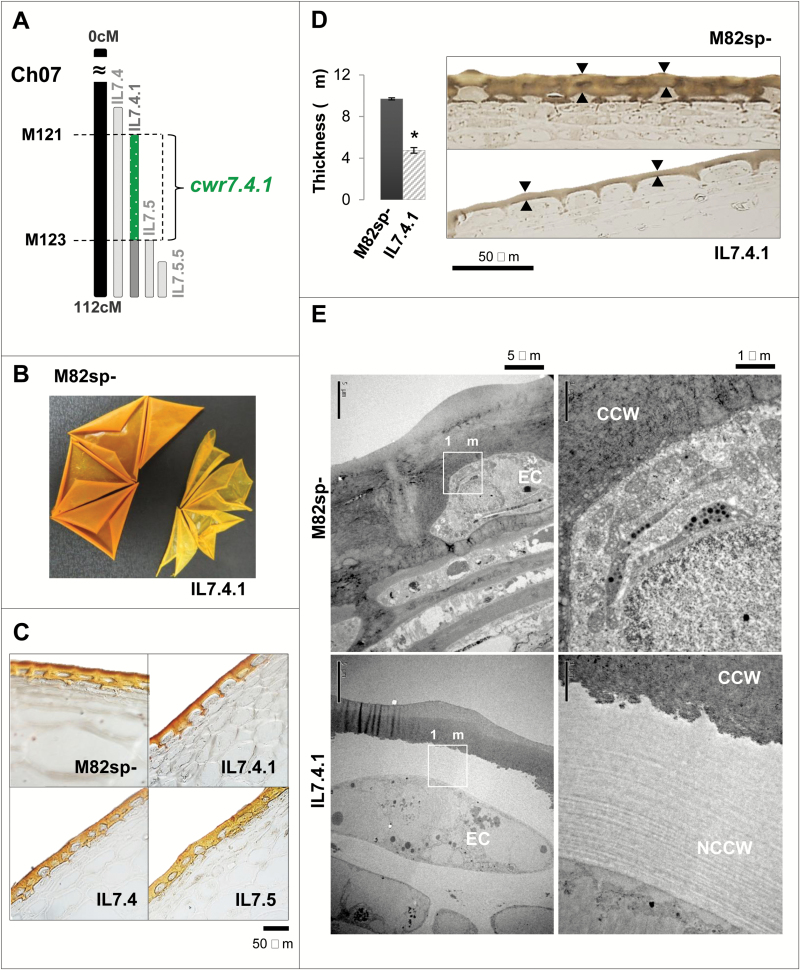
**Putative cell wall-related QTL identified in IL7.4.1 fruits**. **A.** Chromosome intervals. **B.** Macroscopic changes in cuticular membrane phenotype. **C.** Cuticle phenotype under light microscopy using the lipid-specific dye Sudan IV as stain. **D.** Reduction in cuticle thickness (black arrows). Ultrathin sections for TEM stained with lipid-specific osmium tetroxide were used for cuticle thickness measurements. Data are presented as mean±SD. **E.** TEM appearance of changes in the cell wall of the epidermal cell (EC) in mature red tomatoes. CCW, cutinized cell wall; NCCW, non-cutinized cell wall. (This figure is available in colour at *JXB* online.)

The IL population was also phenotyped for cuticle thickness as a proxy for possible changes in fruit cuticular lipids. A significant marker trait association in the SNPs located in the introgression harbored by IL7.4.1 was found that exhibited a very thin, fragile isolated cuticle (Fig. 6B). In fact, IL7.4.1—which in the *S. pennellii* introgression is contained completely within IL7.4 and partially within IL7.5 and IL7.5.5 (Fig. 6A)—displayed a much thinner cuticle than the other overlapping lines, even thinner than the cuticle from parental M82 (Fig. 6C), as revealed by staining with the lipid-specific dye Sudan IV. More precisely, IL7.4.1 showed an almost two-fold reduction in cuticle thickness compared with the parental line (Fig. 6D). However, our analysis revealed that IL7.4.1 fruit did not accumulate fewer waxes or cutin monomers compared with the parental line M82. To investigate other causes of this cuticular phenotype, TEM was used to examine structural differences in more detail. TEM showed that IL7.4.1 contains a layer of non-cutinized cell wall beneath the cuticle, whereas the parental line M82 showed a completely cutinized cell wall (Fig. 6E). This phenotype was also observed in a second-year experiment, suggesting that changes in some cell wall components may influence the effect of IL7.4.1 cuticle thickness without altering its lipid composition.

### Candidate genes for the validated QTLs

Using the online resources available for tomato, a search for candidate genes in the four validated QTLs was performed. Out of the 606 genes found in the four intervals (a total of 3.73 Mb), nine candidate genes can be proposed (Table 2) on the basis of their gene annotation, their changes in gene expression levels in ripe fruit samples from informative ILs containing the significant QTLs compared with the reference background, and their SNP variability between *S. pennellii* and *S. lycopersicum*, which would produce non-conservative protein changes (Supplementary Tables S2 and S3). Thus, in *vlcfa3.4* an ortholog of the Arabidopsis *ECERIFERUM3* gene (*AtCER3*), *Solyc03g117800*, was found. This gene has been described as being involved in the wax biosynthetic decarbonylation pathway (Rowland *et al.*, 2007; Lam *et al.*, 2012) producing the different *n*-aldehydes and *n*-alkanes. The expression levels for this gene on the published databases for the IL population and for the parental *S. pennellii* revealed that it was increased in IL3.4 compared with the parental *S. lycopersicum* M82, but no change was observed for the wild species *S. pennellii* (Supplementary Table S2). In addition to this, six non-conserved amino acid substitutions, including three amino acid insertions (Supplementary Table S3), were found when the protein sequences for both *S. lycopersicum* and *S. pennellii* alleles were compared. For the second QTL in IL3.4, *amy3.4*, no annotated biosynthetic gene likely involved in the production of the amyrins was found. Incidentally, an ortholog of the Arabidopsis *JASMONATE-ZIM-DOMAIN PROTEIN 3* gene (*AtJAZ3*), *Solyc03g118540* (Table 2), was found in this region. This gene has been described as an inhibitor of methyl jasmonate biosynthesis (Valenzuela *et al.*, 2016), involved in the activation of pentacyclic triterpenoid biosynthesis in Arabidopsis (Misra *et al.*, 2014). This gene was found to be down-regulated in IL3.4 compared with the parental M82 (Supplementary Table S2), which fitted with the expected biological effect and, in addition, it contained a non-conservative change in its protein sequence (Supplementary Table S3). Moving on to the next QTL in chromosome 8, *ehfa8.3*, an ortholog of the Arabidopsis *CYP86A8* gene (AtLCR), *Solyc08g081220*, (Table 2) was found. This gene has been described as a fatty acid (ω-1)-hydroxylase, which uses non-epoxidated fatty acids to incorporate a hydroxyl group at the end of the molecule (Kandel *et al.*, 2006). Although no changes in gene expression pattern could be detected (Supplementary Table S2), a non-conservative amino acid change was found in the wild protein (Supplementary Table S3). Finally, when looking for candidate genes in *cwr7.4.1*, four genes with annotations that could be associated with cell wall remodeling processes (Table 2) were identified: two homologs of the Arabidopsis *XYLOGLUCAN ENDOTRANSGLYCOSYLASE 7* gene (*AtXTR7*), *Solyc07g055990* and *Solyc07g056000*, which is involved in secondary cell wall development and organization (Bourquin *et al.*, 2002; Han *et al.*, 2015); an uncharacterized tomato *POLYGALACTURONASE 28*, *Solyc07g056290,* with pectinase activity; and a homolog of the Arabidopsis *SHATTERPROOF 1* gene (*AtSHP1/AGL1*), the *SlTAGL1 Solyc07g055920*, which affects pericarp thickness (Vrebalov *et al.*, 2009). Both xyloglucan endotransglycosilases and the SHATTERPROOF 1 homolog, but not POLYGALACTURONASE 28, showed a reduction in their gene expression levels in IL7.4.1 compared with the expression level in the parental M82 (Supplementary Table S2). For the four candidate genes, non-conservative changes in their predicted amino acid sequence were observed (Supplementary Table S3), especially in POLYGALACTURONASE 28 and SHATTERPROOF1, which contained several deletions in addition to point amino acid substitutions.

## Discussion

Given its complexity, studies aimed at deciphering the genetic basis of cuticular lipid composition in tomato using large populations are scarce (Yeats *et al.*, 2012; Bolger *et al.*, 2014; Ofner *et al.*, 2016), and the situation is not much better for other plants. To date, only one study in tomato has centered on the fruit cuticle and this was mainly focused on wax triterpenoid composition (Yeats *et al.*, 2012); all other studies have targeted leaves. Here, we propose the first screening done for a large set of cuticular lipids (not only amyrins) in tomato fruit using the *S. pennellii* IL population. The wild species *S. pennellii* exhibits great differences in cuticular lipid composition, ranging from higher amounts of wax to reduced cutin monomer coverage, compared with the fruit of cultivated *S. lycopersicum* cv. M82 (Yeats *et al.*, 2012). In fact, *S. pennellii* fruit is characterized by the presence of a thin cuticle showing reduced levels of the major cutin monomer 10-ω-hydroxy hexadecanoic acid but increased levels of the minor cutin monomer 9,10,18-trihydroxy octadecanoic acid. Additionally, the *S. pennellii* cuticle contains higher levels of *n*-alkanes, in particular C_29_ and C_30_*n*-alkanes, but very low levels of α- and δ-amyrins (Yeats *et al.*, 2012). Of these five special cuticular traits, described for the wild accession LA716, in the present study we found that three of them showed significant variation in the IL population: reduced amyrins, reduced cuticle thickness, and increased *n*-alkane levels.

The biosynthesis of *n*-alkanes occurs by the decarbonylation pathway (Riederer and Müller, 2006). *CER3* has been described in Arabidopsis as the main biosynthetic gene involved in this pathway, in association with *CER1* (Rowland *et al.*, 2007; Lam *et al.*, 2012). In *vlcfa3.4* we found the tomato ortholog of the *AtCER3* gene, associated with an increase in *n*-aldehyde and *n*-alkane composition. The putative protein sequence for the *S. pennellii* wild allele contains some non-conservative changes that may affect substrate specificity when compared with the cultivated allele, which might result in stronger activation of the wax decarbonylation pathway and may explain the phenotype observed. Thus, *vlcfa3.4* could represent a strong candidate in the study of CER3 function in the decarbonylation pathway for tomato fruit.

Although the reduction in amyrin content has been described for *S. pennellii*, and we found an already-reported QTL associated with this trait in IL12.1 (*amy12.1*), we also found an additional QTL involved in the contrasting phenotype, that is, the over-accumulation of the three amyrins in *amy3.4*. The metabolic changes observed in *amy3.4* were shared by both IL3.4 and IL3.5, suggesting that the molecular basis associated with this phenotype would be present only in this shared region. As mentioned above, we could not find any amyrin biosynthetic gene in this region. However, we propose the ortholog of *AtJAZ3*, a negative regulator of methyl jasmonate biosynthesis (Valenzuela *et al.*, 2016), as the putative gene involved in the over-accumulation of amyrins. Misra *et al.* (2014) described how methyl jasmonate was involved in the accumulation of amyrins in Arabidopsis. Taking these two reports in mind, we postulate that JAZ3 could also regulate amyrin biosynthesis by regulating methyl jasmonate biosynthesis in tomato fruit. Moreover, the wild allele version of this gene showed a decrease in expression levels in tomato fruit (Supplementary Table S2), together with important changes in its protein sequence (Supplementary Table S3) that could be the reason for the increased amyrin content observed in IL3.4. Further studies of methyl jasmonate content in association with the biosynthesis of amyrins could clarify this type of regulation.

The thinner cuticular phenotype observed in the wild species *S. pennellii* has been associated with changes in the accumulation of the major cutin monomer (Yeats *et al.*, 2012). In contrast with this, here we found a very similar cuticular phenotype, which was independent from the cuticular lipid composition in *cwr7.4.1*. These results differ from those obtained in cuticular mutants in which cutin composition or polymerization was affected (Hovav *et al.*, 2007; Isaacson *et al.*, 2009; Girard *et al.*, 2012; Shi *et al.*, 2012; Petit *et al.*, 2014; Yeats *et al.*, 2014). The most recent studies on tomato fruit cuticle consolidate the idea of the cuticle as a cell wall scaffold over which the different cuticular lipids are distributed (Guzman-Puyol *et al.*, 2015; Segado *et al.*, 2016*a*, 2016*b*). In this context, having a genomic region associated with the interaction between cell wall and CLCs represents a great opportunity to consolidate this working model. One of the genes mapped to *cwr7.4.1* was the tomato gene *SlTAGL1*, which is involved in pericarp thickness (Vrebalov *et al.*, 2009). One possibility could be that this gene might affect cell wall composition not only in pericarp cells but also in epidermal cells. As the *cwr7.4.1* QTL was mapped to IL7.4.1, which completely overlaps with IL7.4, it could be that *SlTAGL1* requires the function of another gene mapped on to the IL7.4.1 region, and only when the alleles of both genes belong to the same species (i.e. both cultivated alleles or both wild alleles) do they show activity. In this context, the presence of several indels and point non-conservative amino acid changes in the wild allele could be essential in the interaction with other proteins encoded by alleles from the same species. Another three genes involved in cell wall remodeling were also mapped on to this QTL. These genes affect the xyloglucan and pectin links with the cellulose and hemicellulose scaffold (Bourquin *et al.*, 2002; Han *et al.*, 2015). Thus, another hypothesis to explain the phenotype observed in *cwr7.4.1* could give details on how defective alleles for these genes might affect the formation of the cell wall scaffold, which would also affect the deposition of cuticular lipids on it. As a result, part of the cell wall surrounding the epidermal cells would be incompletely cutinized, as we saw for IL7.4.1 (Fig. 6E). For either or both hypotheses the detailed characterization of this QTL might be of great interest to elucidate the means by which both types of cuticular components are connected and regulated.

Moreover, we also found a fourth QTL, *ehfa8.3*, which affects the accumulation of epoxyhydroxy fatty acids. Very little is known about the biosynthetic pathway of the epoxyhydroxy fatty acids, and no related biosynthetic gene or regulator has been described in tomato fruit. These types of compounds have been described as being involved in plant defenses against biotic stress such as fungal infection or wounding (Aghofack-Nguemezi *et al.*, 2011). Thus, more in-depth studies on IL8.3 could not only provide clues to decipher such pathways in tomato fruit but also could help to improve traits associated with yield and post-harvest behavior. In *ehfa8.3* we mapped the ortholog to *AtLCR*, whose product which hydroxylates the last carbon of a C_18:X_ fatty acid and rarely uses epoxy fatty acids as a substrate in Arabidopsis (Kandel *et al.*, 2006). One possibility could be that this gene in tomato could take both epoxy- and non-epoxidated fatty acids as a substrate. Another possibility might be that the tomato allele hydroxylates 9,10-dihydroxy fatty acids, which are considered to be the precursors of the epoxyhydroxy fatty acids (Meesapyodsuk and Qiu, 2011). Although we found a non-conservative change in the amino acid sequence of this gene in the wild allele, we did not find a differential expression of this gene for either IL8.3 or *S. pennellii*. One explanation for this finding could be that this gene acts in tomato fruit earlier than the red stage. However, it will be necessary to conduct molecular analyses to clarify the function of this gene in tomato fruit.

In addition to all the above-mentioned traits, the increased levels of trihydroxy fatty acids described for the wild species *S. pennellii* was hardly observed in our experiments with the IL population. Thus, although we observed this phenotype in IL8.3.1 during the first year of experiments, it was not confirmed during the second year as a result of a large variation in the parental line M82. This could suggest that, contrary to the other validated QTLs, this QTL in IL8.3.1 was not strong enough. However, this IL could be a starting point for further studies on trihydroxy fatty acid biosynthesis and/or regulation [we found some promising candidate genes in this IL (data not shown)]. Finally, we were unable to identify any QTL associated with the major cutin monomer, 9,16-dihydroxy hexadecanoic acid, although this monomer was drastically reduced in the *S. pennellii* cuticles. Possible explanations for this finding could be strict homeostatic regulation and/or that the alteration of only one gene in the pathway does not affect the synthesis of this monomer (perhaps some of the genes in the pathway are duplicated), or even that two or more genes (or copies) distantly linked or located on different chromosomes could be altered to produce the diminished accumulation observed in *S. pennellii*. The analysis of populations such as RILs or MAGIC—in which epistatic interactions can be investigated—could shed some light on this trait.

In conclusion, here we present a first comprehensive screening of cuticular lipid composition in a large population. The *S. pennellii* IL population has been shown to be an excellent resource to study the molecular basis of cuticular composition, as suggested by the QTLs mapped and confirmed in this study. Furthermore, some candidate genomic regions and associated genes have been reported in association with some of the main cuticular traits observed for the wild species *S. pennellii*. We therefore believe that our results represent the starting point for future studies using different inbreeding populations, as well as for conducting associated molecular studies to advance our understanding of the molecular basis underlying fruit cuticular phenotypes.

## Supplementary data

Supplementary data are available at *JXB* online.

Table S1. DNA markers used to screen cuticular lipid components in the *S. pennellii* IL population.

Table S2. Gene expression levels for the selected candidate genes.

Table S3. Gene and protein information for the selected candidate genes.

Fig. S1. Validated QTLs associated with the main cuticular lipid traits of *S. pennellii* fruits during the second season.

## Supplementary Material

supplementary_figure_S1_Tables_S1_S3
