# Supplementary material for: Uncovering tomato quantitative trait loci and candidate genes for fruit cuticular lipid composition using the Solanum pennellii introgression line population
Source: J Exp Bot. 2017 May 4;68(11):2703–16. doi: 10.1093/jxb/erx134 (PMC5853253; doi:10.1093/jxb/erx134)
Supplement: supplementary_figure_S1_Tables_S1_S3 [file erx134_suppl_supplementary_figure_s1_tables_s1_s3.pdf]

Journal of Experimental Botany,

Uncovering QTL and candidate genes for tomato fruit cuticular lipid composition using the *Solanum pennellii* introgression line population

Josefina-Patricia Fernandez-Moreno, Dorit Levy-Samoha, Sergey Malitsky, Antonio J. Monforte, Diego Orzaez, Asaph Aharoni, Antonio Granell

SUPPLEMENTAL DATA

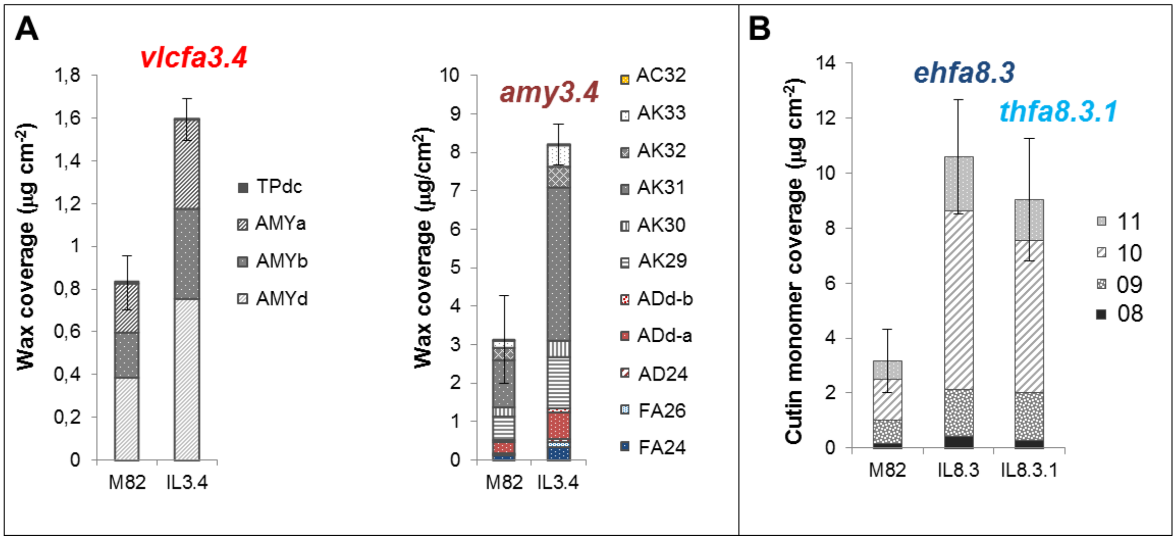

Supplementary Figure S1 Validated QTLs associated with the main cuticular lipid traits of *S. pennellii* fruits during the second season. **A.** Wax related QTLs from IL3.4. **B.** Cutin-related QTLs from IL8.3 and IL8.3.1. Abbreviations: vlcfa, very-long-chain fatty acids; amy, amyrins; ehfa, epoxyhydroxy fatty acids; thfa, trihydroxy fatty acid; AC, alcohol; AD, aldehyde; AK, alkane; FA, fatty acid; ADd, aldehyde derivative; and TPd, triterpenoid derivative. See cutin monomers #08 to #11 in Figure 1. For more genetic marker information see Supplementary Table S1. Error bar, standard error (n = 3).

Supplementary Table S1 DNA markers used to screen cuticular lipid components in the *S. pennellii* IL population.

M#	Genetic Marker	Chromosome	Introgression Line Mapped							IL BIN[a]	Distance MB	Distance bp	Into gene…	Upstream from gene…
1	solcap_snp_sl_15058	ch01	IL1.1.3							1A	0.05	45434	-	Solyc01g005030
2	solcap_snp_sl_15049	ch01	IL1.1.3	IL1.1.2	IL1.1					1A/1B	0.30	303202	Solyc01g005430	-
3	solcap_snp_sl_24801	ch01	IL1.1.3	IL1.1.2	IL1.1					1B	0.66	663658	Solyc01g006000	-
4	solcap_snp_sl_60393	ch01	IL1.1.3	IL1.1.2	IL1.1					1B/1C	1.12	1118868	Solyc01g006540	-
5	solcap_snp_sl_60143	ch01	IL1.1.3		IL1.1					1C	2.51	2507736	Solyc01g008420	-
6	solcap_snp_sl_59964	ch01	IL1.1.3		IL1.1	IL1.2				1C/1D	3.59	3591306	Solyc01g009380	-
7	solcap_snp_sl_20438	ch01	IL1.1.3		IL1.1	IL1.2				1D	3.90	3899743	Solyc01g009660	-
8	solcap_snp_sl_59897	ch01	IL1.1.3		IL1.1	IL1.2				1D/1E	4.40	4404784	Solyc01g009870	-
9	solcap_snp_sl_41438	ch01			IL1.1	IL1.2				1E	45.89	45889601	Solyc01g049690	-
10	solcap_snp_sl_27832	ch01			IL1.1	IL1.2				1E/1F	85.90	85901981	Solyc01g094400	-
11	CL016133-0426_solcap_snp_sl_100368	ch01				IL1.2				1F	86.54	86540312	Solyc01g095180	-
12	solcap_snp_sl_28225	ch01				IL1.2				1F/1G	87.59	87591685	Solyc01g096510	-
13	solcap_snp_sl_28247	ch01					IL1.3			1G/1H	87.70	87703118	Solyc01g096670	-
14	solcap_snp_sl_43902	ch01					IL1.3			1H	88.53	88528976	Solyc01g097830	-
15	solcap_snp_sl_40154	ch01					IL1.3	IL1.4		1H/1I	91.76	91756586	Solyc01g103090	-
16	solcap_snp_sl_40303	ch01					IL1.3	IL1.4		1I	92.48	92478179	Solyc01g104000	-
17	CL016564-0245_solcap_snp_sl_100427	ch01					IL1.3	IL1.4		1I/1J	93.90	93899146	-	Solyc01g105790
18	solcap_snp_sl_54854	ch01						IL1.4		1J	95.75	95746081	Solyc01g108420	-
19	CL017046-0184_solcap_snp_sl_100500	ch01						IL1.4	IL1.4.18	1J/1K	97.10	97104537	Solyc01g109980	-
20	CL016160-0228_solcap_snp_sl_100374	ch01						IL1.4	IL1.4.18	1K	97.89	97893381	Solyc01g111710	-
21	solcap_snp_sl_53612	ch01						IL1.4	IL1.4.18	1K	98.41	98411966	Solyc01g112320	-
22	solcap_snp_sl_70010*	ch02	IL2.1.1	IL2.1						2A	2.17	2166200	-	Solyc02g055550
23	solcap_snp_sl_2559	ch02	IL2.1.1	IL2.1						2A	15.08	15081835	Solyc02g014090	-
24	solcap_snp_sl_7413	ch02	IL2.1.1	IL2.1						2A/2B	25.88	25876791	Solyc02g030450	-
25	solcap_snp_sl_12647	ch02		IL2.1						2B	30.58	30577418	Solyc02g036360	-
26	CL016266-0283_solcap_snp_sl_100390	ch02		IL2.1	IL2.2					2B/2C	35.33	35331701	Solyc02g063270	-
27	solcap_snp_sl_36484	ch02		IL2.1	IL2.2					2C	35.74	35735971	Solyc02g064650	-
28	Le009381_51_solcap-snp-sl-100681	ch02		IL2.1	IL2.2					2C/2D	36.24	36248146	Solyc02g065080	-
29	solcap_snp_sl_15696	ch02			IL2.2					2D	36.79	36790779	Solyc02g065630	-
30	solcap_snp_sl_36373	ch02			IL2.2	IL2.3				2D/2E	36.91	36912602	Solyc02g065770	-
31	solcap_snp_sl_20325	ch02			IL2.2	IL2.3				2E	37.24	37243652	Solyc02g066990	-
32	CL017477-0450_solcap_snp_sl_100578	ch02			IL2.2	IL2.3				2E/2F	37.90	37900875	Solyc02g067790	-
33	solcap_snp_sl_8464	ch02				IL2.3				2F	38.54	38544729	Solyc02g068590	-
34	solcap_snp_sl_33547	ch02				IL2.3	IL2.4			2F/2G	39.24	39240130	Solyc02g069310	-
35	solcap_snp_sl_29779	ch02				IL2.3	IL2.4			2G	43.55	43551066	Solyc02g078930	-
36	solcap_snp_sl_18432	ch02				IL2.3	IL2.4			2G/2H	45.04	45037985	Solyc02g080970	-
37	solcap_snp_sl_18448	ch02				IL2.3	IL2.4	IL2.5		2H	45.32	45323529	Solyc02g081310	-
38	CL009173-0541_solcap_snp_sl_18432	ch02				IL2.3	IL2.4	IL2.5		2H/2I	45.52	45518495	Solyc02g081640	-

39	solcap_snp_sl_4831	ch02					IL2.4	IL2.5			2I	47.22	47218361	Solyc02g083990	-
40	CL016442-0124_solcap_snp_sl_100410	ch02					IL2.4	IL2.5			2I/2J	48.16	48161217	Solyc02g085180	-
41	solcap_snp_sl_36297	ch02						IL2.5			2J	50.57	50566459	Solyc02g088480	-
42	solcap_snp_sl_67138	ch02						IL2.5	IL2.6		2J/2K	51.85	51852193	Solyc02g090230	-
43	solcap_snp_sl_66965	ch02						IL2.5	IL2.6		2K	52.77	52766779	Solyc02g091490	-
44	solcap_snp_sl_58462	ch02						IL2.5	IL2.6	IL2.6.5	2K/2L	53.97	53969561	Solyc02g093010	-
45	solcap_snp_sl_20052	ch02						IL2.5	IL2.6	IL2.6.5	2L	54.11	54107130	Solyc02g093160	-
46	solcap_snp_sl_20051	ch02						IL2.5	IL2.6	IL2.6.5	2L/2M	54.12	54118621	Solyc02g093180	-
47	solcap_snp_sl_47897	ch02							IL2.6	IL2.6.5	2M	55.03	55033465	Solyc02g094510	-
48	solcap_snp_sl_47880	ch02							IL2.6	IL2.6.5	2M	55.24	55238304	Solyc02g094640	-
49	solcap_snp_sl_63048	ch03	IL3.1								3A	0.07	71076	Solyc03g005100	-
50	solcap_snp_sl_63184	ch03	IL3.1								3A	0.75	748576	Solyc03g006060	-
51	solcap_snp_sl_63290	ch03	IL3.1	IL3.2							3A/3B	1.39	1390288	Solyc03g006870	-
52	solcap_snp_sl_26399	ch03		IL3.2							3B	6.40	6396398	Solyc03g034440	-
53	solcap_snp_sl_30006	ch03		IL3.2	IL3.3						3B/3C	11.51	11510772	Solyc03g045090	-
54	solcap_snp_sl_29993	ch03		IL3.2	IL3.3						3C	28.89	28855686	Solyc03g059360	-
55	CL009162-0240_solcap_snp_sl_100138	ch03		IL3.2	IL3.3						3C/3D	60.59	60587533	Solyc03g098230	-
56	solcap_snp_sl_65890	ch03			IL3.3						3D	61.07	61069454	Solyc03g098790	-
57	solcap_snp_sl_58651	ch03			IL3.3	IL3.4					3D/3E	61.63	61632372	Solyc03g110900	-
58	solcap_snp_sl_62473	ch03			IL3.3	IL3.4					3E	63.31	63304984	Solyc03g113000	-
59	solcap_snp_sl_20830	ch03			IL3.3	IL3.4					3E/3F	66.54	66536917	Solyc03g117370	-
60	solcap_snp_sl_9137	ch03				IL3.4					3F	66.72	66720829	Solyc03g117600	-
61	CL015122-0237	ch03				IL3.4	IL3.5				3F/3G	67.22	67226370	Solyc03g118310	-
62	solcap_snp_sl_61677	ch03				IL3.4	IL3.5				3G	67.47	67466637	Solyc03g118620	-
63	solcap_snp_sl_61632	ch03				IL3.4	IL3.5				3G/3H	67.63	67627318	Solyc03g118820	-
64	solcap_snp_sl_33933	ch03					IL3.5				3H	68.83	68825599	Solyc03g120430	-
65	solcap_snp_sl_67626	ch03					IL3.5				3H	70.72	70719370	Solyc03g124010	-
66	solcap_snp_sl_45224	ch04	IL4.1.1	IL4.1							4A	0.04	35288	Solyc04g005030	-
67	solcap_snp_sl_45276	ch04	IL4.1.1	IL4.1							4A	0.32	323820	Solyc04g005480	-
68	solcap_snp_sl_45332	ch04	IL4.1.1	IL4.1							4A/4B	0.59	585828	Solyc04g005910	-
69	solcap_snp_sl_63899	ch04		IL4.1							4B	1.24	1242445	Solyc04g007560	-
70	CL016761-0264_solcap_snp_sl_100460	ch04		IL4.1	IL4.2						4B/4C	1.97	1974622	-	Solyc04g008290
71	solcap_snp_sl_64358	ch04		IL4.1	IL4.2						4C	3.51	3505857	-	Solyc04g010190
72	solcap_snp_sl_41575	ch04		IL4.1	IL4.2						4C/4D	4.94	4942620	Solyc04g014680	-
73	solcap_snp_sl_41609	ch04		IL4.1	IL4.2	IL4.3					4D	5.30	5295726	Solyc04g015130	-
74	solcap_snp_sl_16978	ch04		IL4.1	IL4.2	IL4.3	IL4.3.2				4D/4E	5.53	5529704	Solyc04g015340	-
75	solcap_snp_sl_18763	ch04			IL4.2	IL4.3	IL4.3.2				4E	30.38	30381093	Solyc04g024520	-
76	solcap_snp_sl_23472	ch04			IL4.2	IL4.3	IL4.3.2				4E/4F	55.73	55727496	Solyc04g064550	-
77	solcap_snp_sl_2189	ch04				IL4.3	IL4.3.2				4F	57.78	57782525	Solyc04g070970	-
78	solcap_snp_sl_43402	ch04				IL4.3	IL4.3.2				4F	59.32	59322491	Solyc04g072290	-
79	solcap_snp_sl_47229	ch04						IL4.4			4H	63.22	63226704	Solyc04g078490	-
80	solcap_snp_sl_47615	ch04						IL4.4			4H	64.83	64829060	Solyc04g080730	-
81	solcap_snp_sl_4176	ch04						IL4.4			4H	66.45	66453691	-	Solyc04g083000
82	CL015995-0443_solcap_snp_sl_100344	ch05	IL5.1								5A(1)	0.02	2874	Solyc05g005000	-
83	solcap_snp_sl_69219	ch05	IL5.1								5A(1)	1.08	108012	Solyc05g005100	-
84	solcap_snp_sl_52699	ch05	IL5.1	IL5.2							5A(1)/5B	1.18	1184835	Solyc05g006560	-
85	SGN-U567719_snp239_solcap_snp_sl_52710	ch05	IL5.1	IL5.2							5B	1.21	1211190	Solyc05g00680	-
86	solcap_snp_sl_52714	ch05	IL5.1	IL5.2							5B/5A(2)	1.31	1311252	Solyc05g006650	-
87	solcap_snp_sl_19103	ch05	IL5.1								5A(2)	1.86	1861325	Solyc05g007220	-
88	SGN-U566039_snp997	ch05	IL5.1								5A(2)	2.23	2231292	Solyc05g007770	-
89	solcap_snp_sl_51230	ch05			IL5.3						5D	16.04	16037654	Solyc05g016380	-
90	solcap_snp_sl_55302	ch05			IL5.3						5D	35.40	35397812	Solyc05g025650	-
91	solcap_snp_sl_37413	ch05			IL5.3	IL5.4					5D/5E	63.50	63498544	Solyc05g053400	-

92	solcap_snp_sl_37514	ch05				IL5.4				5E	63.88	63880865	-	Solyc05g053850
93	solcap_snp_sl_224	ch05				IL5.4	IL5.5			5E/5F	64.09	64086547	Solyc05g054120	-
94	solcap_snp_sl_37626	ch05				IL5.4	IL5.5			5F	64.40	64403993	Solyc05g054880	-
95	solcap_snp_sl_306	ch05				IL5.4	IL5.5			5F/5G	64.79	64789300	Solyc05g055010	-
96	solcap_snp_sl_37825	ch05					IL5.5			5G	65.27	65269905	Solyc05g055760	-
97	solcap_snp_sl_37979	ch05					IL5.5			5G	65.77	65770270	Solyc05g056500	-
98	solcap_snp_sl_23100	ch06	IL6.1							6A	0.04	37722	Solyc06g005060	-
99	solcap_snp_sl_31170	ch06	IL6.1							6A	17.07	17071424	Solyc06g018100	-
100	CL017670-0525_solcap_snp_sl_100614	ch06	IL6.1	IL6.2	IL6.2.2					6A/6B	35.73	35725164	Solyc06g052070	-
101	solcap_snp_sl_68935	ch06	IL6.1	IL6.2	IL6.2.2					6B	36.72	36716563	Solyc06g053840	-
102	solcap_snp_sl_68976	ch06	IL6.1	IL6.2	IL6.2.2					6B/6C	37.22	37216563	Solyc06g054440	-
103	solcap_snp_sl_12646	ch06		IL6.2	IL6.2.2					6C	39.29	39287820	Solyc06g062280	-
104	solcap_snp_sl_27215	ch06		IL6.2	IL6.2.2					6C/6D	41.99	41994475	Solyc06g066810	-
105	solcap_snp_sl_39163	ch06		IL6.2						6D	42.56	42561121	Solyc06g065680	-
106	solcap_snp_sl_57574	ch06		IL6.2		IL6.3				6D/6E	44.82	44818656	-	Solyc06g072680
107	solcap_snp_sl_57455	ch06		IL6.2		IL6.3				6E	45.32	45311835	Solyc06g073510	-
108	solcap_snp_sl_57352	ch06		IL6.2		IL6.3				6E/6F	45.95	45953573	Solyc06g074330	-
109	solcap_snp_sl_57228	ch06				IL6.3				6F	46.79	46785478	Solyc06g075370	-
110	solcap_snp_sl_54339	ch06				IL6.3	IL6.4			6F/6G	48.74	48740122	Solyc06g083230	-
111	solcap_snp_sl_54259	ch06				IL6.3	IL6.4			6G	49.10	49096703	Solyc06g083760	-
112	solcap_snp_sl_54183	ch06				IL6.3	IL6.4			6G	49.63	49631131	Solyc06g084530	-
113	solcap_snp_sl_68725	ch07	IL7.1	IL7.2						7A	0.07	68730	Solyc07g005030	-
114	solcap_snp_sl_68398	ch07	IL7.1	IL7.2						7A	1.56	1557551	Solyc07g006690	-
115	solcap_snp_sl_68044	ch07	IL7.1	IL7.2						7A/7B	3.08	3078229	Solyc07g008310	-
116	solcap_snp_sl_15775	ch07		IL7.2						7B	3.21	3207474	Solyc07g008410	-
117	solcap_snp_sl_11082	ch07		IL7.2	IL7.3					7B/7C	3.46	3460132	-	Solyc07g008540
118	solcap_snp_sl_55157	ch07		IL7.2	IL7.3					7C	31.13	31132702	Solyc07g026770	-
119	CL017617-0913_solcap_snp_sl_100601	ch07		IL7.2	IL7.3	IL7.4				7C/7D	59.21	59211604	Solyc07g048010	-
120	solcap_snp_sl_53455	ch07		IL7.2	IL7.3	IL7.4				7D	61.91	61906229	Solyc07g053460	-
121	solcap_snp_sl_55514	ch07		IL7.2	IL7.3	IL7.4	IL7.4.1			7D/7E	63.64	63644286	Solyc07g055700	-
122	solcap_snp_sl_37108	ch07				IL7.4	IL7.4.1			7E	64.74	64739866	Solyc07g061780	-
123	solcap_snp_sl_70925	ch07				IL7.4	IL7.4.1	IL7.5		7E/7F	65.82	65817889	Solyc07g063330	-
124	solcap_snp_sl_70865	ch07				IL7.4	IL7.4.1	IL7.5		7F	66.12	66119424	Solyc07g063750	-
125	solcap_snp_sl_37065	ch07				IL7.4	IL7.4.1	IL7.5	IL7.5.5	7F/7G	66.41	66406912	Solyc07g064130	-
126	solcap_snp_sl_70636	ch07				IL7.4	IL7.4.1	IL7.5	IL7.5.5	7G	67.16	67162714	Solyc07g065370	-
127	solcap_snp_sl_37030	ch07				IL7.4	IL7.4.1	IL7.5	IL7.5.5	7G	67.83	67827948	-	Solyc07g066370
128	CL015795-0319_solcap_snp_sl_100308	ch08	IL8.1.3	IL8.1.1	IL8.1					8A	0.03	28273	Solyc08g005030	-
129	solcap_snp_sl_56672	ch08	IL8.1.3	IL8.1.1	IL8.1					8A	1.67	1667609	Solyc08g007100	-
130	solcap_snp_sl_56922	ch08	IL8.1.3	IL8.1.1	IL8.1					8A/8B	3.00	3003469	Solyc08g008610	-
131	solcap_snp_sl_50349	ch08		IL8.1.1	IL8.1					8B	29.10	29095157	Solyc08g023270	-
132	solcap_snp_sl_4431	ch08		IL8.1.1	IL8.1					8B	53.23	53231057	Solyc08g065320	-
133	solcap_snp_sl_48675	ch08				IL8.2.1	IL8.2			8D	54.69	54694932	Solyc08g066220	-
134	solcap_snp_sl_29394	ch08				IL8.2.1	IL8.2			8D	58.13	58127626	-	Solyc08g069050
135	solcap_snp_sl_25111	ch08				IL8.2.1	IL8.2			8D/8E	61.84	61842932	Solyc08g077960	-
136	solcap_snp_sl_64977	ch08					IL8.2			8E	61.91	61910573	Solyc08g078040	-
137	solcap_snp_sl_65030	ch08					IL8.2	IL8.3		8E/8F	62.13	62132704	Solyc08g078320	-
138	solcap_snp_sl_65244	ch08					IL8.2	IL8.3		8F	63.11	63109885	Solyc08g079630	-
139	solcap_snp_sl_34811	ch08					IL8.2	IL8.3		8F/8G	63.52	63516877	Solyc08g080190	-
140	solcap_snp_sl_34942	ch08						IL8.3		8G	64.63	64630487	Solyc08g081640	-
141	solcap_snp_sl_65565	ch08						IL8.3	IL8.3.1	8G/8H	65.06	65055799	Solyc08g082210	-
142	solcap_snp_sl_70252	ch08						IL8.3	IL8.3.1	8H	65.48	65479618	Solyc08g082800	-
143	solcap_snp_sl_70166	ch08						IL8.3	IL8.3.1	8H	65.84	65836909	Solyc08g083380	-
144	solcap_snp_sl_17547	ch09	IL9.1.2	IL9.1						9A	0.05	48822	Solyc09g005080	-
145	solcap_snp_sl_44783	ch09	IL9.1.2	IL9.1						9A	0.37	369436	Solyc09g005550	-
146	solcap_snp_sl_14679	ch09	IL9.1.2	IL9.1	IL9.1.3					9A/9B	0.74	743707	Solyc09g007100	-
147	solcap_snp_sl_57946	ch09	IL9.1.2	IL9.1	IL9.1.3					9B	1.69	1692427	Solyc09g008240	-

148	CL017729-0357_solcap_snp_sl_100626	ch09	IL9.1.2	IL9.1	IL9.1.3							9B/9C	1.98	1981919	Solyc09g008490	-
149	solcap_snp_sl_39857	ch09		IL9.1	IL9.1.3							9C	2.19	2194152	Solyc09g008770	-
150	solcap_snp_sl_26678	ch09		IL9.1	IL9.1.3	IL9.2.5	IL9.2					9C/9D	2.41	2411368	Solyc09g009080	-
151	solcap_snp_sl_39790	ch09		IL9.1	IL9.1.3	IL9.2.5	IL9.2					9D	3.01	3014917	-	Solyc09g009600
152	solcap_snp_sl_39725	ch09		IL9.1	IL9.1.3	IL9.2.5	IL9.2					9D/9E	3.48	3477979	Solyc09g010080	-
153	CL017345-0677_solcap_snp_sl_100546	ch09		IL9.1	IL9.1.3		IL9.2					9E	4.05	4050706	Solyc09g010740	-
154	solcap_snp_sl_39515	ch09		IL9.1	IL9.1.3		IL9.2					9E/9F	4.56	4561644	Solyc09g011220	-
155	solcap_snp_sl_39407	ch09			IL9.1.3		IL9.2					9F	5.19	5191470	Solyc09g011930	-
156	CL016923-0271_solcap_snp_sl_100489	ch09			IL9.1.3		IL9.2					9F/9G	6.01	6011768	Solyc09g014380	-
157	solcap_snp_sl_51520	ch09					IL9.2					9G	38.36	38363827	Solyc09g055260	-
158	solcap_snp_sl_43215	ch09					IL9.2	IL9.2.6				9G/9H	62.33	62329356	Solyc09g064850	-
159	solcap_snp_sl_43184	ch09					IL9.2	IL9.2.6				9H	62.68	62683510	Solyc09g065010	-
160	solcap_snp_sl_43175	ch09					IL9.2	IL9.2.6				9H/9I	62.99	62985276	Solyc09g065120	-
161	solcap_snp_sl_43011	ch09						IL9.2.6				9I	64.53	64528435	Solyc09g066120	-
162	solcap_snp_sl_46662	ch09						IL9.2.6	IL9.3			9I/9J	66.28	66276116	Solyc09g074480	-
163	solcap_snp_sl_46782	ch09						IL9.2.6	IL9.3			9J	66.93	66926136	Solyc09g075120	-
164	solcap_snp_sl_46828	ch09						IL9.2.6	IL9.3			9J/9K	67.07	67068484	Solyc09g075350	-
165	solcap_snp_sl_3430	ch09							IL9.3			9K	67.69	67687663	Solyc09g076000	-
166	SGN-U320210_C2_At1g04190_snp253	ch09							IL9.3	IL9.3.1	IL9.3.2	9K/9L	68.26	68260072	Solyc09g082540	-
167	solcap_snp_sl_25723	ch09							IL9.3	IL9.3.1	IL9.3.2	9L	69.86	69863659	Solyc09g090350	-
168	solcap_snp_sl_22329	ch09							IL9.3	IL9.3.1	IL9.3.2	9L/9M	70.84	70836949	Solyc09g091580	-
169	solcap_snp_sl_69978	ch09							IL9.3	IL9.3.1		9M	71.64	71641196	Solyc09g092640	-
170	solcap_snp_sl_63588	ch09							IL9.3	IL9.3.1		9M	72.38	72380672	Solyc09g098590	-
171	solcap_snp_sl_45992	ch10	IL10.1									10A	0.05	49125	Solyc10g005040	-
172	solcap_snp_sl_29033	ch10	IL10.1									10A	33.20	33200066	-	Solyc10g045390
173	solcap_snp_sl_33007	ch10	IL10.1		IL10.2							10A/10B	60.49	60491093	-	Solyc10g078790
174	solcap_snp_sl_8260	ch10			IL10.2							10B	60.66	60664352	Solyc10g079010	-
175	solcap_snp_sl_20256	ch10			IL10.2							10B/10C	60.99	60987253	Solyc10g079450	-
176	SGN-U317657_C2_At3g47930_snp417	ch10		IL10.1.1	IL10.2	IL10.2.2						10C/10D	61.02	61017028	Solyc10g079470	-
177	solcap_snp_sl_59259	ch10		IL10.1.1	IL10.2	IL10.2.2						10D	62.01	62007607	Solyc10g080810	-
178	solcap_snp_sl_59474	ch10		IL10.1.1	IL10.2	IL10.2.2						10D/10E	62.74	62741784	Solyc10g081720	-
179	solcap_snp_sl_59494	ch10			IL10.2							10E	62.81	62813840	-	Solyc10g081860
180	solcap_snp_sl_33805	ch10			IL10.2		IL10.3					10E/10F	62.98	62982634	Solyc10g083110	-
181	CL017176-0241_solcap_snp_sl_100516	ch10			IL10.2		IL10.3					10F	63.68	63678125	Solyc10g084000	-
182	solcap_snp_sl_60817	ch10			IL10.2		IL10.3					10F/10G	64.76	64760912	Solyc10g085640	-
183	solcap_snp_sl_60745	ch10					IL10.3					10G	65.01	65011189	Solyc10g086030	-
184	solcap_snp_sl_60643	ch10					IL10.3					10G	65.46	65462812	-	Solyc10g086700
185	solcap_snp_sl_9452	ch11	IL11.1									11A	0.06	57631	Solyc11g005060	-
186	solcap_snp_sl_66431	ch11	IL11.1									11A	1.25	1347455	Solyc11g006740	-
187	solcap_snp_sl_10594	ch11	IL11.1	IL11.2								11A/11B	2.48	2481288	Solyc11g008250	-
188	solcap_snp_sl_20963	ch11	IL11.1	IL11.2								11B	3.65	3652983	Solyc11g010580	-
189	solcap_snp_sl_62695	ch11	IL11.1	IL11.2								11B/11C	4.82	4819599	Solyc11g011880	-
190	solcap_snp_sl_62846	ch11		IL11.2								11C	5.89	5885686	Solyc11g013020	-
191	solcap_snp_sl_6905	ch11		IL11.2	IL11.3							11C/11D	7.84	7843871	Solyc11g017040	-
192	solcap_snp_sl_7444	ch11		IL11.2	IL11.3							11D	25.02	25023476	-	Solyc11g032130
193	solcap_snp_sl_31030	ch11		IL11.2	IL11.3							11D/11E	52.80	52804397	Solyc11g067050	-
194	solcap_snp_sl_32030	ch11			IL11.3							11E	53.50	53502001	Solyc11g068950	-
195	solcap_snp_sl_56104	ch11			IL11.3	IL11.4						11E/11F	54.64	54639039	Solyc11g070100	-
196	solcap_snp_sl_17550	ch11			IL11.3	IL11.4						11F	54.97	54970111	Solyc11g071530	-
197	solcap_snp_sl_44882	ch11			IL11.3	IL11.4						11F/11G	55.15	55148073	Solyc11g071770	-
198	solcap_snp_sl_17563	ch11				IL11.4						11G	55.33	55330360	-	Solyc11g072050

199	solcap_snp_sl_28472	ch11				IL11.4	IL11.4.1			11G/11H	55.58	55584310	-	Solyc11g072420
200	solcap_snp_sl_28504	ch11				IL11.4	IL11.4.1			11H	55.77	55766807	Solyc11g072590	-
201	solcap_snp_sl_17578	ch11				IL11.4	IL11.4.1			11H	56.25	56249720	Solyc11g073200	-
202	solcap_snp_sl_28840	ch12	IL12.1.1	IL12.1	IL12.2					12A	0.05	53649	Solyc12g005100	-
203	solcap_snp_sl_3130	ch12	IL12.1.1	IL12.1	IL12.2					12A	3.77	376932	Solyc12g005700	-
204	solcap_snp_sl_45733	ch12	IL12.1.1	IL12.1	IL12.2					12A/12B	0.72	715204	Solyc12g006170	-
205	solcap_snp_sl_23497	ch12		IL12.1	IL12.2					12B	1.34	1341538	Solyc12g006920	-
206	solcap_snp_sl_45923	ch12		IL12.1	IL12.2					12B/12C	1.70	1695254	-	Solyc12g007290
207	solcap_snp_sl_41031	ch12			IL12.2					12C	2.32	2329931	Solyc12g009030	-
208	solcap_snp_sl_41162	ch12			IL12.2	IL12.3.1	IL12.3			12C/12D	3.03	3034418	Solyc12g009840	-
209	solcap_snp_sl_63520	ch12			IL12.2	IL12.3.1	IL12.3			12D	6.33	6326442	-	Solyc12g016200
210	solcap_snp_sl_40598	ch12			IL12.2	IL12.3.1	IL12.3			12D/12E	8.95	8948057	Solyc12g019000	-
211	solcap_snp_sl_52512	ch12				IL12.3.1	IL12.3			12E	39.99	39993806	-	Solyc12g042530
212	solcap_snp_sl_19344	ch12				IL12.3.1	IL12.3			12E/12F	61.86	61864163	Solyc12g055850	-
213	solcap_snp_sl_42740	ch12					IL12.3			12F	63.01	63006395	Solyc12g056940	-
214	solcap_snp_sl_31966	ch12					IL12.3	IL12.4		12F/12G	64.06	64057242	Solyc12g088840	-
215	solcap_snp_sl_32776	ch12					IL12.3	IL12.4		12G	65.19	65192188	-	Solyc12g096170
216	solcap_snp_sl_6568	ch12					IL12.3	IL12.4		12G/12H	66.31	66305031	Solyc12g098960	-
217	SGN-U567105_snp715_solcap_snp_sl_31389	ch12						IL12.4		12H	66.65	66648008	Solyc12g099530	-
218	SL10284_277_solcap_snp_sl_10131	ch12						IL12.4	IL12.4.1	12H/12I	66.80	66795576	Solyc12g099800	-
219	solcap_snp_sl_31342	ch12						IL12.4	IL12.4.1	12I	66.89	66885302	Solyc12g099930	-
220	solcap_snp_sl_31304	ch12						IL12.4	IL12.4.1	12I	67.03	67033605	Solyc12g100250	-

Data set based on "Tomato - Kazusa and SolCAP markers mapped to genome" (Ofner et al., 2016)
[a] BINs described by Ofner et al. (2016).
* New gene rearrangement in SL2.50 database
DNA markers in black have been described for every IL by Ofner et al. (2016).
DNA markers in blue have been selected herein using the SOLCAP_SNP dataset (Ofner et al., 2016).

Supplemental Table S2 Gene expression levels for the selected candidate genes set.

QTLs[a]	Gene ID	Short name[b]	Gene expression* Parental	Gene expression* Affected ILs			Exp Pat[c]	Diff Exp in S. pen[d]
**(A) - <u>Main QTLs:</u>**								
			**M82**	**IL3.4**	**IL3.5**			
vlcfa3.4 (+)	Solyc03g117800	CER3/WAX2	3.63	4.39	2.55		Comp	No
amy3.4 (+)	Solyc03g118540	TIFY6B/JAZ3	11.87	8.83	14.77		Comp	No
			**M82**	**IL12.1.1**	**IL12.1**	**IL12.2**		
amy12.1 (-)	Solyc12g006520	TTS2	0.14	0.05	0	0	Comp	No
	Solyc12g006530	TTS1	0.23	0.12	0	0	Comp	No
			**M82**	**IL8.3**	**IL8.3.1**			
ehfa8.3 (+)	Solyc08g081220	CYP86A8/LCR	0	0	0		Incomp	No
			**M82**	**IL7.4**	**IL7.4.1**	**IL7.5**		
cwr7.4.1 (-)	Solyc07g056000	XTR7	30.81	16.27	6.9	12.91	Comp	Yes
	Solyc07g055990	XTR7	17.18	8.6	12.48	7.55	Comp	No
	Solyc07g056290	PG8	0	0	0	0	Incomp	No
	Solyc07g055920	TAGL1	163.4	136.14	166.2	120.7	Comp	No

* RPKM values obtained by Illumina RNA-Seq analysis on red tomato fruits from the IL population. Data available in the transcriptome profiling of *S. pennellii* ILs experiment (http://ted.bti.cornell.edu/).

[a] Type of QTL (positive or negative; cutin- or wax-related) is described; ILs containing QTLs are also provided.

[b] Short name based on tomato annotation and similarity to characterized Arabidopsis homologous (see Table 2).

[c] Gene expression pattern. When it is increased or diminished similarly to the metabolite pattern it is considered as compatible (comp). The contrary is considered as Incompatible (Incomp).

[d] Differential gene expression values for *Solanum pennellii* wild parental when compared with *S. lycopersicum*. Based on Supplementary Table sd4 from Koenig et al.. 2013.

Supplemental Table S3 Gene and protein information for the selected candidate genes.

Tomato[a]	Gene ID	Short name	Ch[b]	Position[b]		Strand	Length (bp)	Protein ID	Protein Length	id%[c]	Aln[d]	amino acid substitutions[e]		
				Start	End							Total	Conserv	Non conserv
**(A) Main QTLs:**														
***vlcfa3.4* QTL (600 Kb interval containing 93 genes)**														
S. lyco	Solyc03g117800.2	CER1 protein (Fragment) [AtCER3/WAX2]	3	66830213	66834278	+	4,066	Solyc03g117800.2.1	642					
S. pen	Sopen03g036710	WAX2 C-terminal domain	3	71334247	71338482	+	4,236	Sopen03g036710	644	97.83	630/644	14	8	6 (3 ins)
***amy3.4* QTL (410 Kb interval containing 50 genes)**														
S. lyco	Solyc03g118540.2	Protein TIFY 6B [AtJAZ3]	3	67430375	67433753	+	3,379	Solyc03g118540.2.1	326					
S. pen	Sopen03g037480	tify domain	3	71989456	71993016	+	3,561	Sopen03g037480	326	98.47	321/326	5	4	1
***amy12.1* QTL (0.98 MB interval containing 111 genes)**														
S. lyco	Solyc12g006520.1	SlTTS2: Cycloartenol synthase (Squalene cyclase)	12	1027444	1035605	+	8,162	Solyc12g006520.1.1	698					
S. pen	Sopen12g002480	Prenyltransferase and squalene oxidase	12	1125586	1132992	+	7,407	Sopen12g002480	761	88.94	619/696	43	30	13
S. lyco	Solyc12g006530.1	SlTTS1: Cycloartenol synthase (Squalene cyclase)	12	1041051	1049001	+	7,951	Solyc12g006530.1.1	762					
S. pen	Sopen12g002480	Prenyltransferase and squalene oxidase	12	1125586	1132992	+	7,407	Sopen12g002480	761	98.69	751/761	10	7	3
***ehfa8.3* QTL (540 Kb interval containing 200 genes)**														
S. lyco	Solyc08g081220.1	Fatty acid w-1 hydroxylase [AtCYP86A8]	8	64280043	64281707	-	1,665	Solyc08g081220.1.1	555					
S. pen	Sopen08g029530	Cytochrome P450	8	68842033	68844661	-	2,629	Sopen08g029530	554	98.92	548/554	6	5	1
***cwr7.4.1* QTL (2.18 MB interval containing 263 genes)**														
S. lyco	Solyc07g056000.2	Xyloglucan endotransglucosylase/hydrolase 7	7	63901549	63902824	+	1,276	Solyc07g056000.2.1	288					
S. pen	Sopen07g029350	Glycosyl hydrolases family 16	7	74966267	74967531	+	1,265	Sopen07g029350	287	99.65	286/287	1	0	1
S. lyco	Solyc07g055990.2	Xyloglucan endotransglucosylase/hydrolase 7	7	63891570	63893537	+	1,968	Solyc07g055990.2.1	291					
S. pen	Sopen07g029340	Glycosyl hydrolases family 16	7	74961337	74963344	+	2,008	Sopen07g029340	290	98.62	286/290	4	2	2
S. lyco	Solyc07g056290.1	Polygalacturonase 8 (pectinase)	7	64146906	64150487	+	3,582	Solyc07g056290.1.1	402					
S. pen	Sopen07g029650	Glycosyl hydrolases family 28	7	75225990	75229581	+	3,592	Sopen07g029650	370	90.77	364/401	37	2	35 (31 del)
S. lyco	Solyc07g055920.2	Agamous MADS-box transcription factor (TAGL1)	7	63841344	63851220	+	9,877	Solyc07g055920.2.1	268					
S. pen	Sopen07g029280	K-box region	7	74909447	74919285	+	9,839	Sopen07g029280	265	97.40	262/269	7	0	7 (2 ins/4 del)

[a] Tomato species: cultivated tomato (*Solanum lycopersicum*) and wild tomato (*S. pennellii*)
[b] Ch - chromosome
[c] Data obtained from tomato database SL2.50 (http://www.solgenomics.net/)
[d] id% - percentage of identity between *S. lyco* and *S. pen* proteins obtained by blastp (http://www.solgenomics.net/).
[e] Aln - Protein alignment level: No of conserved amino acids / total of amino acids aligned.
Kb, Kilo bases, MB, Million Bases
